# Interoceptive Awareness of the Breath Preserves Attention and Language Networks amidst Widespread Cortical Deactivation: A Within-Participant Neuroimaging Study

**DOI:** 10.1523/ENEURO.0088-23.2023

**Published:** 2023-06-23

**Authors:** Norman A. S. Farb, Zoey Zuo, Cynthia J. Price

**Affiliations:** 1 Department of Psychology, University of Toronto Mississauga, Mississauga, Ontario L5L 1C6, Canada; 2 Department of Psychological Clinical Sciences, University of Toronto Scarborough, Scarborough, Ontario M1C 1A4, Canada; 3 Department of Biobehavioral Nursing and Health Informatics, University of Washington, Seattle, WA 98195

**Keywords:** attention, awareness, fMRI, interoception, respiration, sensibility

## Abstract

Interoception, the representation of the body’s internal state, serves as a foundation for emotion, motivation, and wellbeing. Yet despite its centrality in human experience, the neural mechanisms of interoceptive attention are poorly understood. The Interoceptive/Exteroceptive Attention Task (IEAT) is a novel neuroimaging paradigm that compares behavioral tracking of the respiratory cycle (Active Interoception) to tracking of a visual stimulus (Active Exteroception). Twenty-two healthy participants completed the IEAT during two separate scanning sessions (*N* = 44) as part of a randomized control trial of mindful awareness in body-oriented therapy (MABT). Compared with Active Exteroception, Active Interoception deactivated somatomotor and prefrontal regions. Greater self-reported interoceptive sensibility (MAIA scale) predicted sparing from deactivation within the anterior cingulate cortex (ACC) and left-lateralized language regions. The right insula, typically described as a primary interoceptive cortex, was only specifically implicated by its deactivation during an exogenously paced respiration condition (Active Matching) relative to self-paced Active Interoception. Psychophysiological interaction (PPI) analysis characterized Active Interoception as promoting greater ACC connectivity with lateral prefrontal and parietal regions commonly referred to as the dorsal attention network (DAN). In contrast to evidence relating accurate detection of liminal interoceptive signals such as the heartbeat to anterior insula activity, interoceptive attention toward salient signals such as the respiratory cycle may involve reduced cortical activity but greater ACC-DAN connectivity, with greater sensibility linked to reduced deactivation within the ACC and language-processing regions.

## Significance Statement

Interoception, the representation of the body’s internal state, is poorly understood compared with the external senses, with existing neuroimaging studies failing to match task difficulty between interoceptive and exteroceptive tasks. The present study used a novel fMRI task to compare interoceptive and exteroceptive attention and explore whether this distinction was moderated by self-reported interoceptive awareness. The results implicate three novel interoceptive mechanisms: interoceptive attention reduces widespread cortical activity while increasing prefrontal connectivity, wherein greater self-reported interoceptive awareness is linked to preserved activation of the anterior cingulate cortex (ACC) and language regions. Rather than increasing activation of interoceptive cortex, interoceptive attention to the breath may involve attending to body representations typically ignored in favor of exteroceptive information and other forms of cognition.

## Introduction

Interoception, the sense of the body’s internal state, is central to human experience, providing homeostatic cues (Strigo and Craig, 2016) that inform emotion (Wiens, 2005; Barrett, 2017), motivation (Craig, 2003; Critchley and Garfinkel, 2017), and wellbeing (Farb et al., 2015;; Chen et al., 2021; Paulus and Khalsa, 2021). Yet the neural mechanisms underlying interoceptive attention are still poorly understood when compared with the five “canonical” human senses (Chen et al., 2021). For example, visual attention enhances the neural response to visual stimuli in well-characterized representation cortices (Liu et al., 2005), and similar progress has been made in characterizing neural representations for hearing (Alho et al., 2014), touch (Puckett et al., 2017), smell (Plailly et al., 2008), and taste (Veldhuizen et al., 2007). Yet despite promising anatomic characterization of a putative interoceptive pathway (Craig, 2002, 2009), how interoceptive attention modulates the neural representation of the body’s internal state remains unclear.

Our understanding has been limited by a focus on interoceptive accuracy by the research community. For example, heartbeat detection paradigms model the ability to detect a liminal cardiac signal (Garfinkel et al., 2015), i.e., a weak biological signal that can easily be missed or confused so that variation in accuracy can be observed. Accuracy paradigms have been productive: superior detection accuracy is linked to greater activation of the anterior cingulate (ACC; Critchley et al., 2004) and anterior insula (Y.W. Chong et al., 2015), hubs of the brain’s “salience network” (SLN; Seeley et al., 2007; Chand and Dhamala, 2016). Yet SLN recruitment may indicate a broader error-monitoring system that does not distinguish interoception from other sensory processes (Baltazar et al., 2021). It is therefore unclear whether the SLN is necessary involved in all interoceptive processes, such as the representation and regulation of salient viscerosomatic signals that are typical challenges in the navigation of everyday emotional experience (Frijda, 2005).

Research confirms that interoceptive accuracy may not be especially relevant for emotion regulation and wellbeing. Clinical populations often show normal interoceptive accuracy (Desmedt et al., 2022), and clinically-efficacious interoception-focused interventions such as mindfulness training do not improve accuracy, despite reliable enhancements in self-reports of interoceptive attention tendency (Parkin et al., 2014; Khalsa et al., 2020). Conversely, while heartbeat detection accuracy can be enhanced through biofeedback training (Meyerholz et al., 2019), such training, in the absence of a broader focus on attentional habits, have yet to be linked to improved wellbeing. Instead, patients with anxiety or panic disorders have historically demonstrated superior interoceptive accuracy (Ehlers and Breuer, 1992; Zoellner and Craske, 1999), but tend to catastrophize interoceptive experience (Domschke et al., 2010). The complex relationship between interoceptive accuracy and wellbeing demonstrates the need to expand the scope of interoceptive research to other clinically relevant constructs, such as how and when interoceptive attention is engaged (Farb et al., 2015).

Interoceptive attention tendency, and specifically the ability to sustain interoceptive attention remains a compelling if underexplored determinant of mental health (Ainley et al., 2016). Improving the capacity to skillfully attend to interoceptive cues remains a central target of contemplative interventions such as mindfulness training (Gibson, 2019; Price and Weng, 2021). Such interventions address intolerance for interoceptive signals, which otherwise leads vulnerable individuals into patterns of experiential avoidance (Anestis et al., 2007; Hayes et al., 1996). Interoceptive sensibility, how one appraises interoceptive signals, shows stronger links to wellbeing than interoceptive accuracy (Ferentzi et al., 2019; Schuette et al., 2021), with a growing literature relating trust in interoceptive signals to greater wellbeing (Calì et al., 2015; Trevisan et al., 2019).

Interoceptive attention seems to recruit a distinct neural network from exteroception that features the middle insula (Haruki and Ogawa, 2021), consistent with its proposed role as a bridge to the prefrontal cortex from primary representation cortices in the posterior insula and somatosensory regions (Pollatos et al., 2007; Craig, 2009; Farb et al., 2013a). Activity in the middle insula characterizes wellbeing (Nord et al., 2021), with hypoactivation linked to depression (Farb et al., 2010, 2022; Avery et al., 2014), and hyperactivation linked to anxiety (Kerr et al., 2016; Tan et al., 2018). Breath monitoring in particular appears to strengthen coherence across a frontotemporal-insular network (Herrero et al., 2018).

Yet neuroimaging studies characterizing interoceptive attention to the breath have been confounded by nonequivalent task demands. Farb et al. (2013a) contrasted exteroceptive and interoceptive attention by comparing a visual condition requiring behavioral responses against passive breath monitoring; Wang et al. (2019) compared a more difficult breath-monitoring task against an easier visual task. In both cases, the more demanding task recruited the dorsal attention network (DAN), while the easier/passive task implicated the default mode network (DMN), confounding conclusions on interoceptive representation.

We therefore developed a novel fMRI paradigm for characterizing the neural dynamics of breath-focused interoception compared with a closely balanced exteroception task. We hypothesized (H1) that interoception would result in greater somato-insular recruitment than exteroception, but reduced DAN activation. We further hypothesized (H2) that greater self-reported interoceptive sensibility would correlate with greater SLN activity during interoception, consistent with the SLN’s ascribed role in sensory monitoring and integration (Menon and Uddin, 2010). We also explored (H3) a paced-breathing condition to understand impact of endogenous versus exogenous respiratory control, and (H4) functional connectivity differences between Interoception and Exteroception.

## Materials and Methods

### Experimental design

This fully within-participant study was conducted to validate a novel interoceptive attention task as part of an NIH-funded pilot study, a two-group randomized control trial to examine the neural correlates of interoceptive awareness in the context of mindful awareness in body-oriented therapy (MABT) training (www.ClinicalTrials.gov, identifier: NCT03583060). MABT is a well-validated, clinical intervention that focuses on developing the interoceptive capacities of identifying, accessing, and appraising internal bodily signal to support adaptive emotion regulation (Price and Hooven, 2018). To maximize power, data from the study’s two assessment timepoints were combined into a single dataset, with group and time included in all statistical models as nuisance covariates.

### Participants

Twenty-two right-handed study participants (11 male and 11 female), of adult age (mean: 36.1 years, range: 18–62) completed both baseline and postintervention assessments. Eleven participants (50% of the sample) were randomly allocated to receive eight MABT sessions, delivered individually once per week for eight weeks. Twenty participants self-identified as White or European American, one as African American, and two as Hispanic. Their highest education levels were high school (*n* = 5), two years of college (*n* = 2), Bachelor’s degree (*n* = 8), and Master’s degree or higher (*n* = 7).

Sample size for the study was determined by simulation-based power analysis. Given the difficulties in a priori registration of all fMRI contrasts, power analysis was conducted to determine minimum sample size for up to 10 focal contrasts while maintaining familywise power ≥0.90; this required a per-test power of $\sqrt[{10}]{\mathrm{.90}}=\mathrm{.9895}$. The α level was determined by taking the typical peak Z required for peak voxel familywise error correction (Z = 4.53), computing its equivalent *p*-value from the normal distribution (*p = *2.95 × 10^−6^), and then Bonferroni correcting this value for 10 comparisons (*p = *2.95 × 10^−7^). The simulated effect size was determined by computing the average of the peak Z scores from all significant clusters revealed by the [Interoception – Exteroception] in Farb et al. (2013a), as were the variances of the contrast main effect and the participant random effects (Z = 5.87, *s^2^_contrasteffect_* = 0.20*, s^2^_randomeffect_* = 0.22). Monte Carlo simulation conducted in the R statistical environment suggested that up to 10 contrasts would be sufficiently powered at *N* ≥ 9, so the parent trial sample size of *N* = 44 scans adequately powered the study design. The script for the power analysis is available on the Open Science Framework (https://osf.io/k6uen).

Twenty-five healthy individuals with self-reported elevated stress were initially recruited through advertisements in a local newspaper and through the University of Washington research volunteer website and flyers posted on campus. Inclusion criteria were: (1) being over 18 years of age; (2) Perceived Stress Scale (Taylor, 2015) scores indicating moderate stress levels; (3) naive to mindfulness-based approaches (no prior experience); (4) agrees to forgo (nonstudy) manual therapies (e.g., massage) and mind-body therapies (e.g., mindfulness meditation) for 12 weeks (baseline to post-test); (5) fluent in English; (6) can attend MABT and assessment sessions; and (7) right-handed (for uniformity of neuroimaging results). Exclusion criteria were: (1) lifetime diagnosis of mental health disorder; (2) unable to complete study participation (including planned relocation, pending inpatient treatment, planned extensive surgical procedures, etc.); (3) cognitive impairment, assessed by the Mini-Mental Status Exam (MMSE; Folstein et al., 1975) if demonstrated difficulty comprehending the consent; (4) use of medications in the past 30 d that affect hemodynamic response; (5) lifetime head injuries or loss of consciousness longer than 5 min; (6) currently pregnant; or (7) contraindications for MRI, e.g., claustrophobia, metal objects in body, etc. The full CONSORT diagram is available online (https://osf.io/76wsj).

### Ethics statement

All participants provided informed consent. The study procedures were reviewed and approved by the institutional review board at the University of Washington in accordance with the World Medical Association Declaration of Helsinki.

### The Interoceptive/Exteroceptive Attention Task (IEAT)

**Figure 1.**
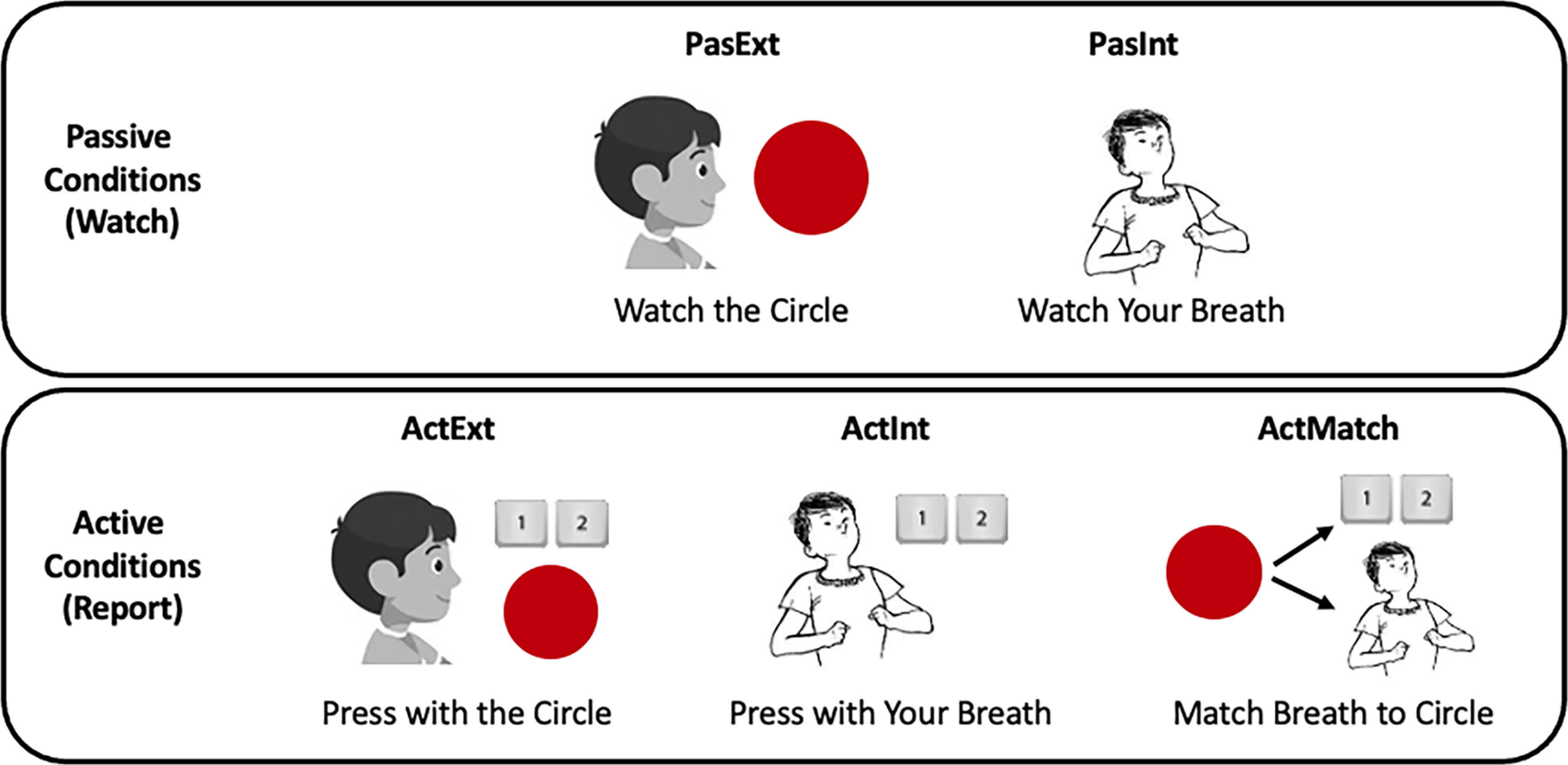
Schematics for the Interoceptive/Exteroceptive Attention Task (IEAT). In Exteroceptive conditions, participants attended to a circle expanding and contracting; in Interoceptive conditions, participants attended to their respiratory inhalation and exhalation. In Passive conditions, participants simply observed the circle or their breath. In Active conditions, participants pressed buttons to track the circle or breath. In the Matching condition, participants tracked the circle’s movements while synchronizing their respiration cycle with the circle. PasExt = Passive Exteroception; PasInt = Passive Interoception; ActExt = Active Exteroception; ActInt = Active Interoception; ActMatch = Active Matching.

The Interoceptive/Exteroceptive Attention Task (IEAT) is a novel paradigm for exploring the neural dynamics of respiratory attention and awareness. The IEAT consisted of five conditions: Passive Exteroception, Passive Interoception, Active Interoception, Active Exteroception, and Active Matching (a paced breathing condition), as shown in Figure 1.

#### Passive conditions

During Passive Exteroception, participants were asked to visually monitor a circle as it expanded and contracted periodically on the MRI-compatible visual display without making any behavioral responses. The circle’s pulse frequency was set to match participants’ in-scanner breathing frequency, obtained from a respiration belt worn by participants in the scanner (usually ∼12 Hz). During Passive Interoception, participants viewed a stationary circle on the screen while attending to sensations of the breath.

#### Active conditions

During Active Exteroception, participants reported on the expansion and contraction of the circle on the screen, which again was set to pulse at participants’ in-scanner respiratory frequency. During Active Interoception, participants reported on inhalations and exhalations by making button box key presses with their right-hand index and middle fingers respectively. The circle on the screen expanded and contracted with these key presses, approximating the frequency of circle movement during Passive Exteroception. During Active Matching, participants reported on the expansion and contraction of the circle as in Active Exteroception, while synchronizing their inhalation to the circle’s expansion and their exhalation to the circle’s contraction.

### Interoceptive attention tendency

Perhaps the most common dispositional index of subjective interoceptive engagement is the Multidimensional Assessment of Interoceptive Awareness (MAIA), which in validation has demonstrated strong associations to subjective wellbeing (Mehling et al., 2012, 2018). The MAIA is a 32-item, multifaceted, self-report questionnaire designed to canvas the five conceptual domains of Interoceptive Awareness, Reactions to Sensation, Attention Regulation, Mind Body Integration, and Trusting Body Sensations (Mehling et al., 2022). The ensuing eight subscales probe experiential domains: Noticing, Body Listening, and Emotional Awareness; regulatory domains: Attention Regulation, Self-Regulation, and Trusting; and problematic reactions: Not Worrying and Not Distracting. The MAIA is sensitive to treatment effects from clinically efficacious interoceptive-focused interventions, with studies demonstrating a positive relationship between improved interoceptive awareness on the MAIA and treatment health outcomes (Fissler et al., 2016; Price et al., 2019; Roberts et al., 2021).

Here, the MAIA was used to provide a subjective report of the ability to adaptively engage interoceptive attention. The MAIA has an updated version to improve the reliability of the two reverse-coded subscales (Mehling et al., 2018), but as the new version was not available at study outset, we followed prior work (Ferentzi et al., 2021), which suggested deriving a total score from the original version was employed while omitting the two problematic subscales (“Not Worrying” and “Not Distracting”). In the present study, this total score yielded good reliability (Cronbach’s α = 0.85). For more complete descriptive statistics and exploration of the MAIA subfactors, please refer to Extended Data Tables 4-1 and 4-2.

### Procedures

Participants completed the IEAT during fMRI acquisition at two timepoints, baseline and three-month follow-up. The MAIA self-report questionnaire was administered to assess interoceptive awareness at both timepoints. Training effects are the subject of separate reports.

### Data analysis

#### Respiration confounds

Changes in breathing depth and rate modulate CO_2_ concentration in the bloodstream, with faster, shallower breathing increasing blood oxygen level-dependent (BOLD) signals throughout the brain (Birn et al., 2006). Failure to correct for task-related variation in breathing rate and depth can therefore lead to spurious associations with experimental conditions (Weiss et al., 2020). Consensus on correction for such effects is an area of ongoing investigation, but fMRI analysis should at a minimum correct for respiratory frequency and respiratory volume/time (RVT; Power et al., 2020).

#### Respiration frequency

Respiration data were acquired using a MR-compatible respiration belt sampling at 500 Hz (Philips model 452213117812). Respiration data were first smoothed using a 1 s zero phase low-pass filter window and then mean-corrected. Breath frequency was then estimated using a fast Fourier transform (FFT) of the respiration period. As a first level covariate, frequency was estimated using a 10 s sliding window across the time series, generating a frequency value for each volume acquired in the timeseries. Trial-specific frequencies were also estimated across each task period to serve as covariates at the second (group) level of analysis.

#### Respiration volume/time

The respiratory signal is influenced by changes in respiratory volume in addition to frequency, with respiratory volume/time (RVT) predicting widespread changes in BOLD activity (Birn et al., 2006). Respiratory belt measurements indicate stretch amplitude rather than volume, but such measurements have been found to be highly correlated with medical grade continuous spirometer measurements, the “gold standard” for measuring respiratory volume (Chu et al., 2019).

To correct for RVT influence, belt amplitude signal was used to calculate RVT as recently recommend (Power et al., 2020) by first finding peaks and troughs of the normalized (z-scored) respiratory signal, setting a minimum distance between peak and trough of 0.5 standard deviations, but a minimum distance of 2 s between consecutive peaks or troughs. Linear interpolation of peaks and troughs generated an “envelope” around the respiratory signal, and the difference between peak and trough values represented the RVT score across the timeseries. Trial-averaged RVTs were also estimated for each task period for use as covariates at the second (group) level of analysis. The envelopes generated for all runs are visualized on the OSF site.

#### Stimulus and behavior timeseries

The three active localizer conditions, Active Exteroception, Active Interoception, and Active Matching required button-presses to track the sensory target, i.e., inhalation/exhalation during the respiratory cycle, or expansion/contraction during the visual circle cycle. Each active condition therefore produced three periodic timeseries: (1) the circle radius, as measured by timestamps in the participant log files, (2) respiration belt amplitude, as measured by pressure transduction on an MR-compatible respiration belt, and (3) a waveform generated from participant button presses, with button presses indicating inflection points (peaks and troughs) of the response waveform.

**Figure 2.**
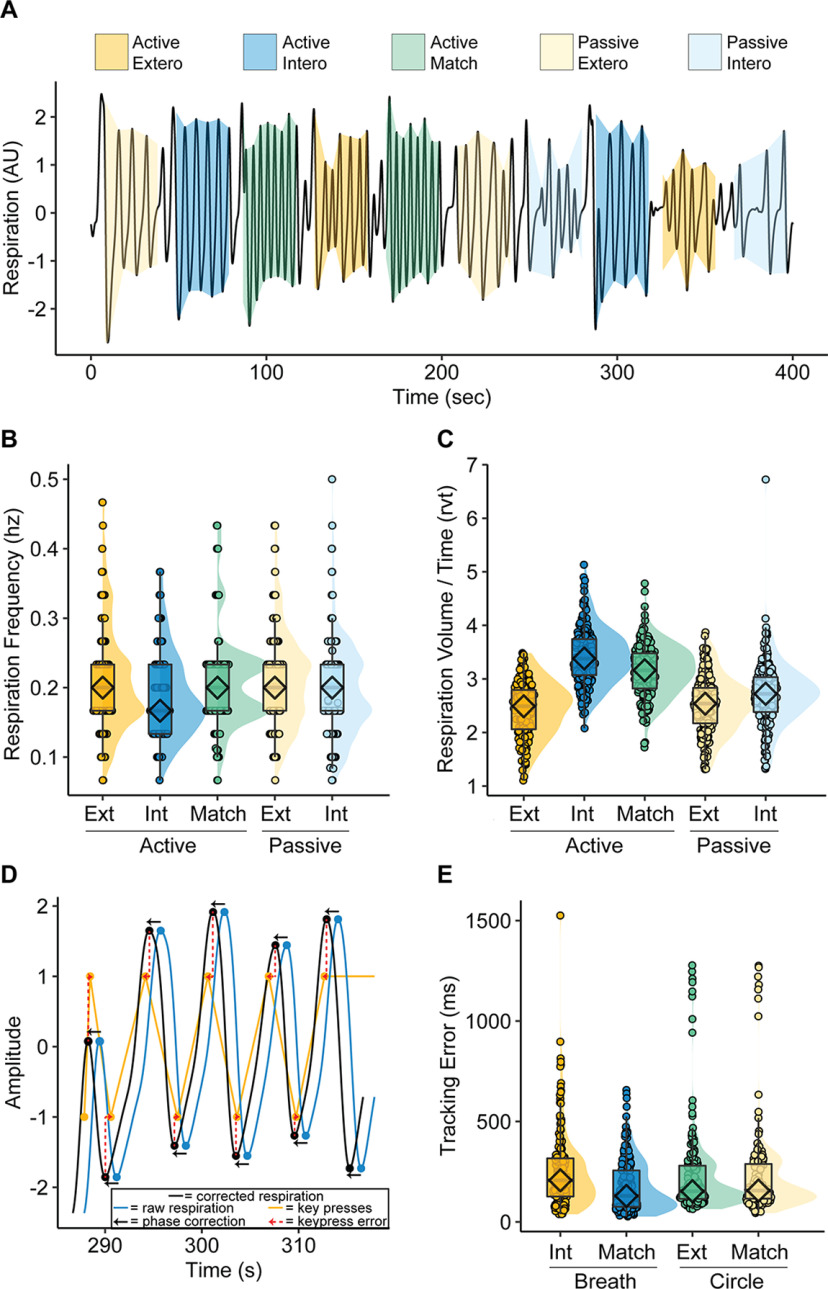
Respiration and behavior tracking. ***A***, Respiratory peak and trough detection was successful for each of the experimental task conditions, allowing for calculation of respiratory frequency (Hz) and respiratory volume over time (RVT). ***B***, Respiration was slower during Active Interoception than in other conditions. ***C***, Respiration was deeper (greater RVT) during Active Interoception and Active Matching than in other conditions. ***D***, To assess tracking accuracy, the respiration waveform (blue) was first temporally adjusted (black) to match phase with keypress signals (orange). The average error (red) between key press timing and respiratory peaks/troughs was calculated; the same procedure was applied to a waveform describing circle radius in the exteroceptive tracking conditions. ***E***, No differences in tracking accuracy were observed between Active Interoception of the Breath and Active Exteroception of the Circle, nor with but Active Matching to the Circle; however, breath tracking was superior during the Active Matching condition. The inhibitory effects of RVT on BOLD activity at the first level of analysis are presented in Extended Data Figure 2-1. Ext/Extero = Exteroception; Int/Intero = Interoception.

The respiration belt waveform was obtained directly from a physiological logfile for each fMRI run. Respiration data were sampled at 500 Hz and included a scanner-generated timestamp to precisely indicate the beginning and end of the functional run. Respiration data were segmented into task trials using timestamps from participant logfiles. The ‘pracma’ library (Borchers, 2021) in the R statistical programming environment (R Core Team, 2017) was used to find peaks and troughs within the timeseries (Fig. 2*A*). The minimum peak difference was set to the sampling rate, as individual breaths were unlikely to have a period shorter than 1 s.

10.1523/ENEURO.0088-23.2023.f2-1 Extended Data Figure 2-1 Effects of the respiratory volume/time (RVT) covariate on BOLD activity. Contrasts maps were generated from the five-condition model corrected using threshold-free cluster estimation (TFCE) to the familywise *p* < 0.05 level. Areas in blue demonstrate reduced BOLD activity with greater levels of RVT. Download Figure 2-1, TIF file.

Behavioral keypress waveforms were generated using linear interpolation of behavioral logfile timestamps for button presses using the *approx* function in R. The inhalation/expansion button “1” was coded as −1 to indicate beginning from the trough of the period, and the exhalation/contraction button “2” was coded as 1 to indicate peak of the period. As the waveforms were intended to be compared with the respiratory signal, the waveforms were interpolated at a sampling rate of 500 Hz to match the data acquired by the respiration belt.

The circle stimulus waveform was obtained from the stimulus presentation software. The circle period was matched to participant-specific breathing frequencies obtained via respiration belt during structural MRI acquisition. For each trial, the timeseries began at an arbitrary unit of 0. The expansion/contraction period was symmetrical and defined as half the participant respiratory period. The circle value was then extrapolated as increasing from −1 for the expansion period to reach a value of 1 by the end of the period; the circle then paused for 0.2 * the expansion period; the circle then decreased at the same rate for the contraction period to reach a value of −1 by the end of the period, and then paused again for 0.2 * the contraction period, repeating the cycle the end of the task block.

#### Tracking accuracy

To calculate trial-specific tracking accuracy in the active tracking conditions (Active Interoception, Active Exteroception, and Active Matching), the appropriate sensory stimulus waveform (respiration in Active Interoception, the visual circle in Active Exteroception) was first phase-corrected to maximally align with the keypress waveform; this correction served to compensate for systematic signal transduction lags between the three types of signals. Phase correction was performed using a normalized cross-correlation (NCC) analysis using the ‘dtwclust’ library in R to estimate the maximum correlation between behavior and respiration/circle timeseries (Sardá-Espinosa, 2019). The NCC analysis tested correlation as function of lag between two timeseries, up to a full second (500 samples) of lag.

Following phase correction, tracking accuracy was assessed by computing the deviation from the respiration/circle inflection points to the nearest keypress, as tracking keypresses were cued by changes in circle/respiration phase (the switch from expansion/inhalation to contraction/exhalation). The average deviation in milliseconds was then calculated for each trial and used as a measure of tracking accuracy, with lower scores indicating more accurate tracking (Fig. 2*D*, below). The complete algorithm for generating timeseries waveforms and calculating tracking accuracy is available on the Open Science Framework (https://osf.io/cuz6s).

#### Neuroimaging data acquisition

Neuroimaging was performed using a 3T Philips Achieva scanner (Philips Inc.) at the Diagnostic Imaging Sciences Center, University of Washington. Imaging began with the acquisition of a T1-weighted anatomic scan (MPRAGE) to guide normalization of functional images (∼6 min) with TR = 7.60 ms, TE = 3.52 ms, TI = 1100 ms, acquisition matrix = 256 × 256, flip angle = 7°, shot interval = 2530 ms, and 1-mm isotropic voxel size. Functional data were acquired using a T2∗-weighted echoplanar imaging (EPI) sequence with TR = 2000, TE = 25 ms, flip angle α = 79°, field of view = 240 × 240 × 129 mm, 33 slices, and a voxel size of 3 × 3 × 3.3 mm with 3.3-mm gap. Button presses were registered using a two-button MR-compatible response pad.

#### Preprocessing

A set of preprocessing steps was performed using the consortium-developed fMRIprep robust preprocessing pipeline for fMRI data (https://fmriprep.readthedocs.io/en/stable/). Briefly, preprocessing consisted of realignment and unwarping of functional images, slice timing correction, and motion correction. The functional images were resliced using a voxel size of 2 × 2 × 2 mm and smoothed using a 6-mm full-width at half-maximal (FWHM) isotropic Gaussian kernel.

#### First-level analysis

Within-participant statistical models were used to characterize the neural distinction between task conditions. Participant time series data from the IEAT was submitted to separate first-level general linear statistical models using Statistical Parametric Mapping software (v12). Task-specific boxcar stimulus functions were convolved with the canonical hemodynamic response function, separately modeling the onsets of the interoceptive and visual control conditions for each participant. To control for motion and physiological confounds, six standard movement parameters, the root mean square of temporal change (DVARS), framewise displacement, respiration rate, and RVT were all included as nuisance covariates.

#### Second-level analysis

Participant first-level maps for each experimental condition [Passive Interoception, Passive Exteroception, Active Interoception, Active Exteroception, Active Matching] were analyzed at the second level using a full-factorial mixed-model ANOVA in SPM12 (Friston, 2007). The second-level contrasts subdivided the tasks in two main effects and their interaction term: Reporting Demand [Active vs Passive] × Target [Interoception vs Exteroception]. Follow-up comparisons within Active [Active Interoception vs Active Exteroception vs Active Matching] and within Passive [Passive Interoception vs Passive Exteroception] were also modelled.

Familywise control for multiple comparisons (corrected *p *<* *0.05) in whole-brain analyses was achieved through threshold-free cluster enhancement (TFCE), which controls familywise error rate based on a permutation testing approach and determines optimal voxel-wise cluster-forming thresholds using an automated algorithmic method (S. Smith and Nichols, 2009). The algorithm eliminates the need to choose between arbitrary correction thresholds by sampling across statistically equivalent peak and cluster size thresholds. Trial-averaged respiration frequency and RVT were modelled at the second level as nuisance covariates.

#### Trial-level confounds

The current study comes from an exploratory clinical trial, the results of which are the subject of a separate report. We combined data across the trial to power the comparison of IEAT task conditions and modelled any effects of trial Group (MABT vs Control), Time (Baseline vs Postintervention) and their interactions as nuisance covariates. All models also contained condition-averaged respiration rate and RVT as covariates, to further control for variation in respiration between experimental conditions. *Post hoc* analyses that did not include the Group and Time covariates did not meaningfully change the reported results.

#### Region of interest (ROI) analysis

For region of interest (ROI) analysis, all signal extractions were taken from models containing the nuisance covariates. Using the built-in SPM12 function, the median value of the raw, unwhitened signal was extracted from all voxels within the ROI, yielding one value per participant at each scanning session. These values were entered into a linear mixed-effects model with restricted likelihood estimation was applied using the ‘lme4’ library (Bates et al., 2015) in the R statistical programming environment.

#### Hypothesis testing

Hypothesis 1 aimed to compare interoceptive and exteroceptive attention. To this end we first evaluated a whole-brain interaction between reporting demand [active vs passive] and attentional target [interoception vs exteroception] to evaluate whether the effects of attentional target should be evaluated separately for the active and passive reporting conditions. Subsequent analyses compared the simple effects of attentional target within each reporting demand condition, i.e., [Passive Interoception vs Passive Exteroception] and [Active Exteroception vs Active Interoception]. To perform these contrasts, all five task conditions were estimated separately at the first (individual session) level of analysis and entered into a full factorial design in SPM12.

**Figure 3.**
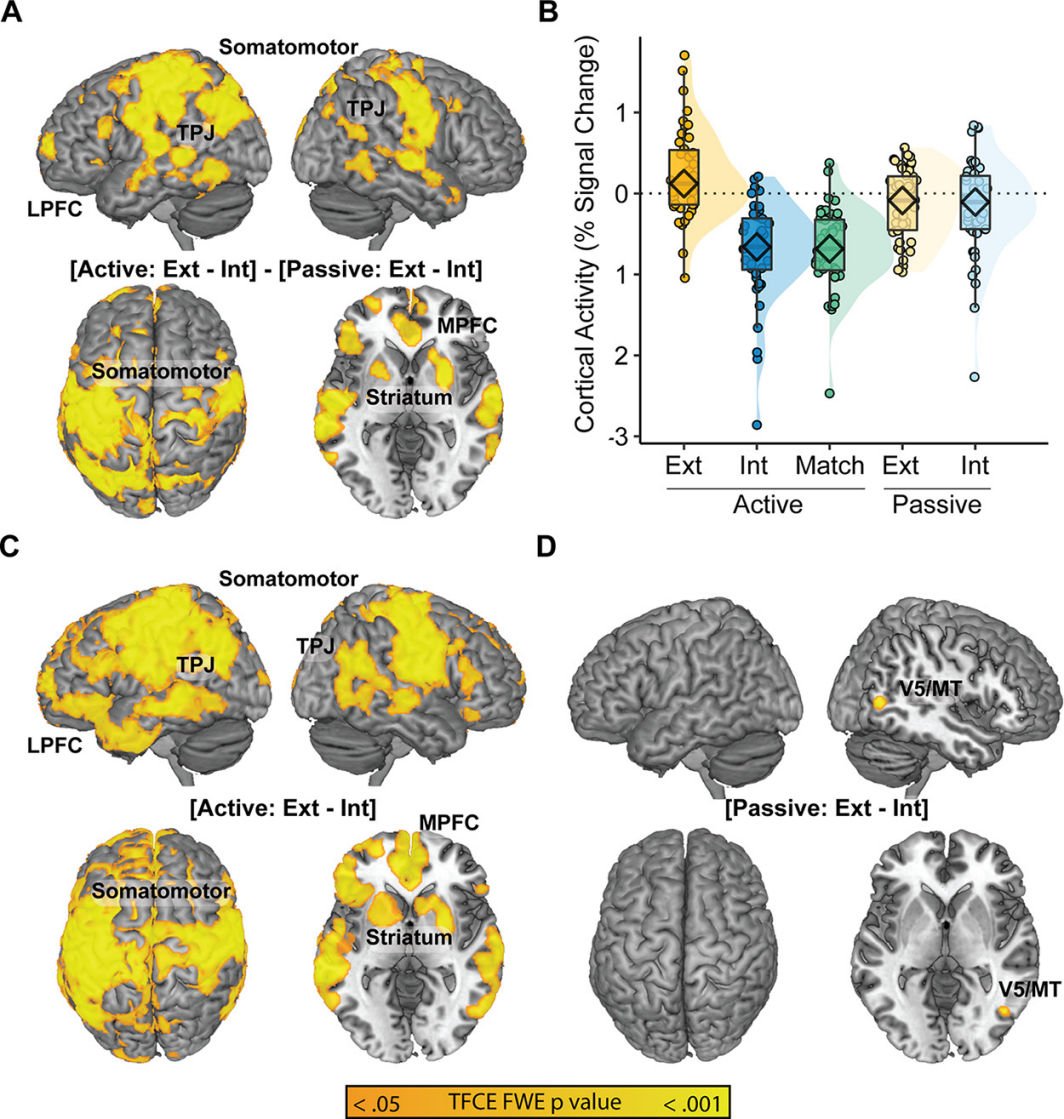
Interactions and simple effects of task. ***A***, Interoceptive Reporting Demand (Active vs Passive) × Attentional Target (Exteroception vs Interoception) implicated the medial and lateral prefrontal cortices (MPFC and LPFC), somatomotor cortices, striatum, and temporal/occipital cortices. ***B***, Median % signal change across the regions revealed highest activation in the Active Exteroception condition and the two Passive conditions relative to Active Interoception and Act Match. ***C***, Simple effects comparisons between Active Exteroception and Interoception conditions largely replicated the interaction effect, suggesting that the Active conditions were driving the interaction. ***D***, Conversely, only area V5/MT distinguished Passive Exteroception from Passive Interoception. Ext = Exteroception; Int = Interoception.

**Figure 4.**
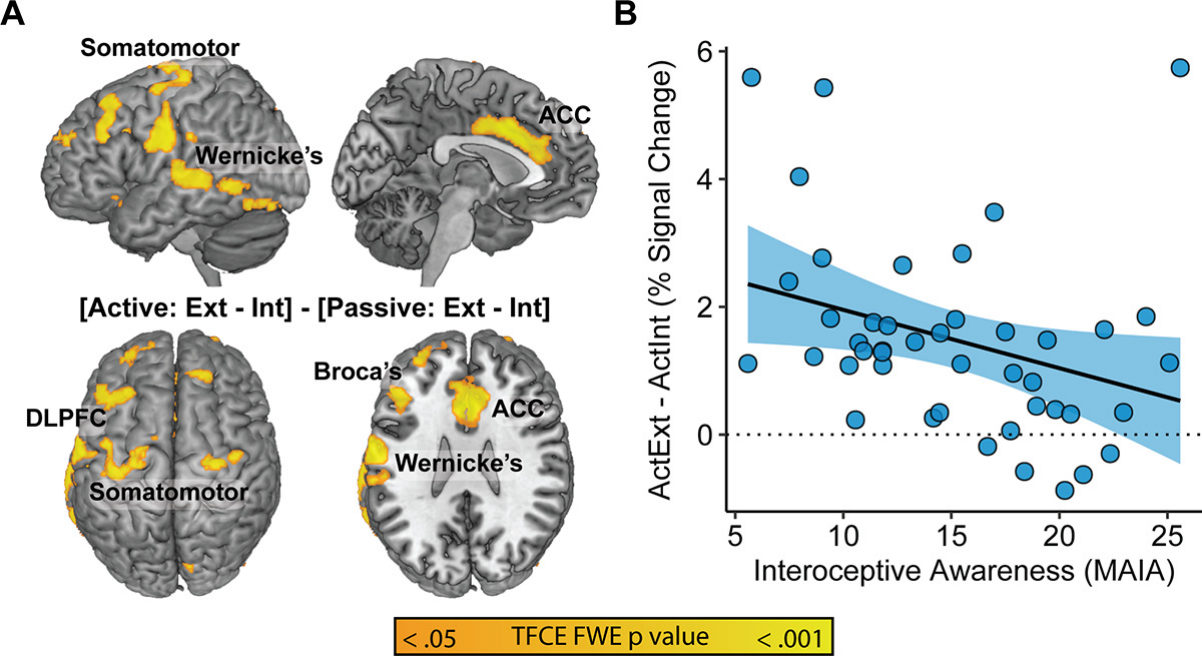
Covariates of self-reported interoceptive awareness. ***A***, Orange regions denote positive correlates of MAIA scores within the [ActExt – ActInt] contrast. ***B***, Scatterplot of the relationship between MAIA score and median neural activity during the [ActExt – ActInt] contrast across significant covariate regions. A more detailed scatterplot showing all five experimental conditions is available in Extended Data Figure 4-1, whole-brain maps of MAIA subfactors as covariates are available with TFCE-correction in Extended Data Figure 4-2, and at a more exploratory (*p* < 0.005, k ≥ 200) threshold in Extended Data Figure 4-3. MAIA scale subfactor intercorrelations and reliabilities are available in Extended Data Table 4-1, factor loadings of these subfactors on a single factor solution are available in Extended Data Table 4-2, and correlations between the subfactors and the Anterior Cingulate Cortex region are available in Extended Data Table 4-3.

Hypothesis 2 aimed to investigate whether the differences between exteroception and interoception were moderated by individual differences in subjective interoceptive awareness (MAIA scores). Focusing on the contrast of [Active Interoception vs Active Exteroception], we first created contrast maps at the first (within session) level of analysis. These first level maps were then entered into a second (group)-level analysis that included normalized (z-scored) MAIA scale total scores as a covariate of interest. The MAIA covariate was subjected to TFCE correction in the same fashion as other whole-brain analyses, and respiration rate and RVT change between the two conditions was included in the factorial model as a nuisance covariate to control for variation associated with physiological changes. Exploratory analyses of each MAIA subfactor are also available as Extended Data Tables 4-1, 4-2, 4-3, and Figures 4-2 and 4-3.

Hypothesis three tested the potential moderating factor of endogenous versus exogenous control of the respiratory cycle by contrasting each of Active Exteroception and Active Interoception against Active Matching using the same five condition model from testing Hypothesis 1.

Hypothesis four aimed to understand how engaging in Active Interoception changes brain connectivity relative to Active Exteroception. To accomplish this aim, a psychophysiological interaction (PPI) analysis was conducted in SPM12 using the Generalized PPI Toolbox (v. 13.1), which improves on standard PPI analyses by estimating the effect each task condition has on connectivity independently (McLaren et al., 2012). Here, we used the model employed in Hypotheses 2, a second (group)-level full factorial model that contained first level contrasts of [Active Interoception – Active Exteroception] and the MAIA covariate term.

To define a region of interest (ROI), the conjunction of the [Active Exteroception – Active Interoception] contrast and the positive MAIA contrast was evaluated, using a threshold of *p* <* *0.001 for each contrast, resulting in a conjoint probability comparable to conservative FWE correction of *p *<* *1 × 10^−6^. The largest cluster observed was used as a seed region, K = 794 voxels, peak Z = 4.06, *x* = −4, *y* = 58, *z* = 14, consistent with dorsal anterior cingulate cortex (ACC; Brodmann area 24). At the first level of analysis, mean time course activity extracted from this seed region was convolved with separate boxcar regressors indicating the onsets and durations of the Active Exteroception and Active Interoception conditions, and the ensuing whole-brain maps were then contrasted to model the PPI effect for each participant session. These first level PPI maps were then collected and analyzed using the same full factorial modeling approach described above.

#### Test-retest reliability

The fact that each participant was scanned twice offered a unique opportunity to test the reliability of study effects across two independent scanning sessions. A conjunction analysis for the four main contrasts reported in this paper was conducted. As the TFCE algorithm does not currently perform conjunction analysis, and halving the sample size reduces experimental power, the baseline and postintervention sessions were each analyzed separately at *p* < 0.0011^/2^ = *p* < 0.0316, so that the resulting overlap would yield a conjunction *p* < 0.001; a minimum cluster size of k ≥ 500 was also applied to each estimate as this cluster size resulted in a FWE corrected *p* value < 0.05.

### Data and code availability

The full study protocol was preregistered with the Open Science Framework (https://osf.io/y34ja). All study materials, including behavioral data and fMRI signal extractions, the code to run the experiment and subsequent fMRI analysis, power analysis, statistics, and graphing scripts, are freely available on the Open Science Framework (https://osf.io/ctqrh/).

## Results

### Control analyses/manipulation checks

Analysis of the control variables (respiratory frequency, RVT, and tracking error) is summarized in Figure 2. The peak and trough detection algorithm (Fig. 2*A*) allowed for RVT estimation (for images of every run in the study, see https://osf.io/ugje4).

### Effects of attention on respiration rate and respiratory volume*/*time (RVT)

The study-wide average respiration frequency was 0.21 Hz (Table 1). A main effect of task condition was observed, *F*_(4,854)_ = 9.36, *p* < 0.001, with follow-up pairwise comparisons suggesting that respiration rate was slower during Interoception conditions than Exteroception conditions, with an average reduction of 0.02 Hz, 95% confidence interval (CI) [0.01, 0.03] (Fig. 2*B*). However, respiration rate was not slower during Exteroception conditions than in the Active Matching condition (Table 2).

**Table 1:** Estimated marginal means for respiration frequency

				95% CI	
Condition	Mean	SE	df	Lower	Upper
Active Interoception	0.188	0.0116	24.6	0.164	0.211
Passive Interoception	0.197	0.0116	24.6	0.173	0.221
Active Matching	0.209	0.0116	24.6	0.185	0.233
Active Exteroception	0.212	0.0116	24.6	0.188	0.235
Passive Exteroception	0.212	0.0116	24.6	0.189	0.236

Data are displayed in Hz, SE = standard error of the estimate, df = degrees of freedom, CI = confidence interval.

**Table 2:** Tukey-adjusted pairwise comparisons of respiration frequency

Contrast			Estimate	SE	df	*t* value	*p* value
ActExt	–	ActInt	0.024	0.00507	854	4.745	<0.0001*
ActExt	–	ActMatch	0.002	0.00507	854	0.449	0.9916
ActExt	–	PasExt	−0.001	0.00507	854	−0.187	0.9997
ActExt	–	PasInt	0.015	0.00507	854	2.921	0.0294*
ActInt	–	ActMatch	−0.022	0.00507	854	−4.296	0.0002*
ActInt	–	PasExt	−0.025	0.00507	854	−4.932	<0.0001*
ActInt	–	PasInt	−0.009	0.00507	854	−1.824	0.3601
ActMatch	–	PasExt	−0.003	0.00507	854	−0.636	0.9692
ActMatch	–	PasInt	0.013	0.00507	854	2.472	0.098
PasExt	–	PasInt	0.016	0.00507	854	3.108	0.0166*

ActExt = Active Exteroception; ActInt = Active Interoception; ActMatch = Active Matching; PasExt = Passive Exteroception; PasInt = Passive Interoception. * indicates *p* values < .05.

Condition had a large impact on RVT, *F*_(4,854)_ = 109.7, *p* < 0.001 with equivalent RVT only between the two exteroception conditions (Table 3). Follow-up analyses confirmed that participants breathed more deeply during Passive Interoception than Exteroception, even more deeply during Active Matching, and deepest during Active Interoception (Fig. 2*C*; Table 4).

**Table 3:** Estimated marginal means for respiration volume/time (RVT)

				95% CI	
Condition	Mean	SE	df	Lower	Upper
Active Exteroception	2.43	0.0407	360	2.35	2.51
Passive Exteroception	2.51	0.0407	360	2.43	2.59
Passive Interoception	2.72	0.0407	360	2.64	2.80
Active Matching	3.15	0.0407	360	3.07	3.23
Active Interoception	3.42	0.0407	360	3.34	3.50

Data are reported in respiratory volume/time, in terms of respiratory belt stretch (arbitrary units) integrated over time.

**Table 4:** Tukey-adjusted pairwise comparisons of respiration volume/time (RVT)

Contrast			Estimate	SE	df	*t* value	*p* value
ActExt	–	ActInt	−0.99	0.0573	854	−17.268	<0.0001*
ActExt	–	ActMatch	−0.713	0.0573	854	−12.44	<0.0001*
ActExt	–	PasExt	−0.078	0.0573	854	−1.361	0.6527
ActExt	–	PasInt	−0.289	0.0573	854	−5.046	<0.0001*
ActInt	–	ActMatch	0.277	0.0573	854	4.828	<0.0001*
ActInt	–	PasExt	0.912	0.0573	854	15.907	<0.0001*
ActInt	–	PasInt	0.701	0.0573	854	12.222	<0.0001*
ActMatch	–	PasExt	0.635	0.0573	854	11.079	<0.0001*
ActMatch	–	PasInt	0.424	0.0573	854	7.394	<0.0001*
PasExt	–	PasInt	−0.211	0.0573	854	−3.685	0.0023*

Data are reported in respiratory volume/time, in terms of respiratory belt stretch (arbitrary units) integrated over time. ActExt = Active Exteroception; ActInt = Active Interoception; ActMatch = Active Matching; PasExt = Passive Exteroception; PasInt = Passive Interoception. * indicates *p* values < .05.

A frequency/RVT trade-off was also evident as negative within-participant correlations, *r*(878) = −0.452, 95% CI [−0.535, −0.434], *p* < 0.001, which may offset the impact of changing respiration rates on BOLD activity. Nevertheless, to control for the influence of condition on respiration, block-specific respiration frequency and RVT were included as covariates in all second-level analyses. Controlling for frequency made had little impact on BOLD activation in subsequent analysis, but greater RVT was associated with widespread cortical deactivation (Extended Data Fig. 2-1), reinforcing the importance of controlling for RVT during data analysis.

### Interoceptive and exteroceptive tracking accuracy

Participant tracking of the sensory stimuli (respiration and visual circle) was analyzed in terms of error (in milliseconds) between key presses and the peaks and troughs of stimulus waveforms within each of the active task blocks. Stimulus waveforms were first phase-corrected to maximally match peaks and troughs with keypresses. Error was then measured as the average time difference between stimulus peaks/troughs and key presses (Fig. 2*D*).

Participants accurately and reliably tracked both the respiratory and visual stimuli (Fig. 2*E*; Table 5) with no error differences between breath tracking in Active Interoception, circle tracking in Active Exteroception, or circle tracking in Active Matching (Table 6). Respiration alignment with keypad responses was superior in Active Matching compared with all other conditions. These results suggest the IEAT successfully matched tracking difficulty between the Active Interoception and Active Exteroception conditions.

**Table 5:** Estimated marginal means for target tracking error

					95% CI	
Target	Condition	Mean	SE	df	Lower	Upper
Breath	Matching	179	28.5	28	120	237
Circle	Matching	231	28.5	28	172	289
Circle	Exteroception	239	28.5	28.1	180	297
Breath	Interoception	262	28.6	28.3	204	321

Target indicates the target for which button tracking accuracy is calculated; for Active Exteroception, this is always the circle, and for Active Interoception, this is always the breath, but for the Active Matching condition, both targets are required to align with keypresses, so both accuracies can be computed. Data reported are in milliseconds lag relative to the target inflection points.

**Table 6:** Tukey-adjusted pairwise comparisons of target tracking error

Target	Condition		Target	Condition	Estimate	SE	df	*t* value	*p* value
Circle	Exteroception	vs	Circle	Match	7.93	17.1	673	0.463	0.967
Breath	Interoception	vs	Circle	Exteroception	23.68	17.3	673	1.372	0.5176
Breath	Interoception	vs	Circle	Match	31.61	17.2	673	1.834	0.2583
Breath	Match	vs	Breath	Interoception	−83.79	17.2	673	−4.861	<0.0001*
Breath	Match	vs	Circle	Exteroception	−60.11	17.1	673	−3.508	0.0027*
Breath	Match	vs	Circle	Match	−52.17	17.1	673	−3.05	0.0127*

Data reported are in milliseconds lag relative to the target inflection points. * indicates *p* values < .05.

### Effects of attentional and reporting demand

A whole-brain interaction analysis between Reporting Demand [Active vs Passive] and Attentional Target [Exteroception vs Interoception] implicated the sensorimotor, temporoparietal, striatum, and prefrontal cortex (Fig. 3*A*); follow-up analysis of the median signal across all significant voxels revealed deactivation in the Active Interoception and Active Matching conditions relative to the other task conditions (Fig. 3*B*; Table 7).

**Table 7:** Task interactions and simple effects

		Cluster	Peak *p*	Peak	Peak	Peak *p*	Coordinates (MNI)		
Description	Side	size (k)	(FWE)	TFCE	Z	(raw)	*x*	*y*	*z*
Interaction [ActExt > ActInt] > [PasExt > PasInt]									
Somatomotor, middle temporal, superior frontal, temperoparietal junction	-	34501	0.001	2389.64	3.54	<0.001	−50	−32	56
Occipital pole	L	231	0.037	1181.47	3.24	0.001	−4	−94	30
Angular gyrus	R	226	0.043	1136.62	3.16	0.001	58	−54	20
Insula	L	169	0.043	1134.44	3.09	0.001	−22	8	−2
Inferior frontal	R	70	0.046	1113.62	3.09	0.001	50	24	18
ActExt > ActInt									
Somatomotor, middle temporal, superior frontal, temperoparietal junction	-	57088	0.001	2540.6	3.54	<0.001	−50	−32	56
Occipital pole	L	318	0.032	1249.39	2.95	0.002	−22	−94	12
Occipital pole	R	55	0.043	1152.73	3.16	0.001	16	−96	16
PasExt > PasInt									
Area V5/MT	R	62	0.033	1057.65	3.09	0.001	46	−64	4

ActExt = Active Exteroception; ActInt = Active Interoception; ActMatch = Active Matching; PasExt = Passive Exteroception; PasInt = Passive Interoception.

A follow-up simple effects contrast of [Active Exteroception > Active Interoception] largely replicated the interaction effect (Fig. 3*C*); conversely, the contrast of [Passive Exteroception > Passive Interoception] revealed only a small cluster of activation in motion-related lateral occipital area V5/MT (Fig. 3*D*). The V5/MT finding is consistent with a failure to fully match task features, as Passive Interoception was the only condition in which the circle stimulus remained stationary rather than pulsing. Given a lack of other distinctions between the passive monitoring conditions, subsequent analyses focused on the active tracking conditions.

### Covariates of self-reported interoceptive awareness

To better understand the nature of the deactivation observed during Active Interoception relative to Active Exteroception, a focal analysis was conducted to investigate the potential moderating role of self-reported interoceptive awareness (MAIA scores). Interoceptive awareness was significantly related to the level of deactivation observed across a subset of the cortical regions implicated in interoception-related deactivation (Fig. 4). Specifically, activity in the anterior cingulate cortex (ACC), dorsomedial prefrontal cortex, and left lateralized language-related regions (e.g., Broca’s and Wernicke’s areas) demonstrated significant associations with interoceptive awareness, such that greater MAIA scores were associated with reduced deactivation during Active Interoception relative to Active Exteroception (Table 8). This pattern was also more generally replicated across both passive and Active Matching conditions (Extended Data Fig. 4-1).

**Table 8:** Positive covariates of MAIA scale on [ActExt > ActInt] contrast

		Cluster	Peak *p*	Peak	Peak	Peak *p*	Coordinates (MNI)		
Description	Side	size (k)	(FWE)	TFCE	Z	(raw)	*x*	*y*	*z*
Anterior cingulate	L	1205	0.025	1677.36	2.85	0.002	−4	12	36
Postcentral (Somatosensory)	L	921	0.03	1594.48	2.79	0.003	−60	−10	32
Precentral (motor)	R	122	0.036	1502.99	2.88	0.002	28	−14	78
Dorsolateral PFC/Broca’s	L	489	0.037	1480.51	2.99	0.001	−34	32	56
Lateral occipital/Wernicke’s	L	1047	0.038	1467.38	2.95	0.002	−52	−78	−20
Lateral occipital	R	160	0.039	1464.02	2.88	0.002	54	−76	−12
Dorsomedial PFC	R	61	0.042	1424.77	3.09	0.001	20	46	54
Insula	L	87	0.045	1386.84	2.71	0.003	−42	−8	−8
Cerebellum	R	77	0.046	1381.72	2.91	0.002	54	−74	−36
Cerebellum	R	74	0.047	1367.92	2.88	0.002	38	−44	−40
Dorsolateral PFC	L	119	0.047	1367.11	2.79	0.003	−30	54	30
Lateral occipital (Superior)	R	32	0.047	1362.52	2.71	0.003	10	−82	44
Cerebellum	R	35	0.048	1358.1	2.95	0.002	24	−46	−50
Frontal pole	R	26	0.048	1355.7	2.64	0.004	26	70	10
Orbitofrontal	L	13	0.048	1351.04	2.73	0.003	−56	24	−10

ActExt = Active Exteroception; ActInt = Active Interoception; ActMatch = Active Matching; PasExt = Passive Exteroception; PasInt = Passive Interoception.

10.1523/ENEURO.0088-23.2023.f4-1 Extended Data Figure 4-1 MAIA scale and anterior cingulate (ACC) activation with all five task conditions. The relationship between interoceptive sensibility/awareness scores (MAIA) and anterior cingulate activity across all five task conditions. Signal was extracted from each experimental condition and plotted above, demonstrating the same reduced separation between conditions, including within the passive conditions. ActExt = Active Exteroception, ActInt = Active Interoception, ActMatch = Active Matching, PasExt = Passive Exteroception, PasInt = Passive Interoception. Download Figure 4-1, TIF file.

10.1523/ENEURO.0088-23.2023.f4-2 Extended Data Figure 4-2 MAIA covariates of the [Active Interoception – Active Exteroception] contrast, corrected using threshold-free cluster estimation (TFCE) to the familywise *p* < 0.05 level. The analysis was run on each MAIA subscale in place of the total score as a covariate in a model that also controlled for study design (Group × Time), respiration frequency, and RVT. Only covariates with significant voxels are displayed; only Emotional Awareness and Self-Regulation showed evidence of covariation. Emotional Awareness also showed a TFCE-corrected cluster of activity in the left planum temporal/superior temporal gyrus. Self-Regulation also showed a TFCE-corrected cluster of activity in the right lateral cerebellum at the border of Crus1 and Crus2. Download Figure 4-2, TIF file.

10.1523/ENEURO.0088-23.2023.f4-3 Extended Data Figure 4-3 MAIA covariates of the [Active Interoception – Active Exteroception] contrast, corrected using the joint thresholds of *p* < .005 and cluster size k ≥ 200 voxels. The analysis was run on each each MAIA subscale in place of the Total score as a covariate in a model that also controlled for study design (Group x Time), respiration frequency, and RVT. Only covariates with significant voxels are displayed. As illustrated above, many components shared substantial overlap with the pattern observed in the MAIA total score. Emotion Awareness, Self-Regulation, Body Listening, and to a lesser extent Attention Regulation and Noticing all showed similar patterns of covariation, a pattern that aligns with the ACC ROI correlations described in Table 4-3. These whole brain analyses do suggest that Emotion Awareness, Self-Regulation, and Body Listening also implicate the left lateralized language regions, suggesting a particular role of the “Mind-Body Integration” conceptual domain in supporting the covariation effect. Noticing only implicated the temporal and somatosensory association aspects of the total score covariate, and conversely, Attention Regulation implicated the ACC without many of the left-temporal language regions. There was little overlap with Trusting, which only implicated the left frontal pole and right cerebellum (Crus 1), Not Worrying, which only implicated the right superior occipital cortex, and no covariation with Not Distracting even at this exploratory threshold. Download Figure 4-3, TIF file.

10.1523/ENEURO.0088-23.2023.t4-1 Extended Data Table 4-1 MAIA Scale subfactor intercorrelations and reliabilities. Download Table 4-1, DOCX file.

10.1523/ENEURO.0088-23.2023.t4-2 Extended Data Table 4-2 Factor loadings for a single factor solution of the eight MAIA subfactors. Download Table 4-2, DOCX file.

10.1523/ENEURO.0088-23.2023.t4-3 Extended Data Table 4-3 Univariate Correlations of Anterior Cingulate ROI with MAIA total score and subscales. Download Table 4-3, DOCX file.

MAIA subscales generally showed comparable correlations with the region of interest (ROI) as the total score (*r* = −0.32 to −0.27) with weaker correlations for Nonjudging and Noticing (*r* ≈ −0.15; Table 9). Emotion Awareness, Self-Regulation, Body Listening showed similar patterns of whole-brain covariation as the total score. Attention Regulation implicated the ACC but not language areas, whereas Noticing implicated the left lateralized language regions but not the ACC. Not Worrying and Not Distracting failed to significantly moderate the deactivation effect, even at more liberal exploratory thresholds.

**Table 9:** MAIA subscale correlates with MAIA covariate ROI: means, SDs, and correlations with confidence intervals

Variable	M	SD	Correlation	95% CI
Total score	15.13	5.35	−0.32*	[−0.56, −0.03]
Nonjudging	26.73	7.59	−0.16	[−0.43, 0.15]
Noticing	2.82	1.07	−0.14	[−0.42, 0.17]
Attention regulation	2.06	1.07	−0.27	[−0.52, 0.03]
Emotion awareness	3.22	0.96	−0.32*	[−0.56, −0.02]
Self-regulation	2.37	1.10	−0.28	[−0.53, 0.02]
Body listening	1.77	1.20	−0.28	[−0.53, 0.02]
Trusting	2.88	1.25	−0.27	[−0.52, 0.03]

M and SD are used to represent mean and standard deviation, respectively. Values in square brackets indicate the 95% confidence intervals. * indicates correlations with *p* values < .05.

As the largest and most powerfully moderated cluster, the ACC region, k = 1205, *x* = −4; *y* = 12; *z* = 36, was retained as a seed ROI in the PPI analysis (H4) described below.

### Endogenous versus exogenous sources of interoceptive control

**Figure 5.**
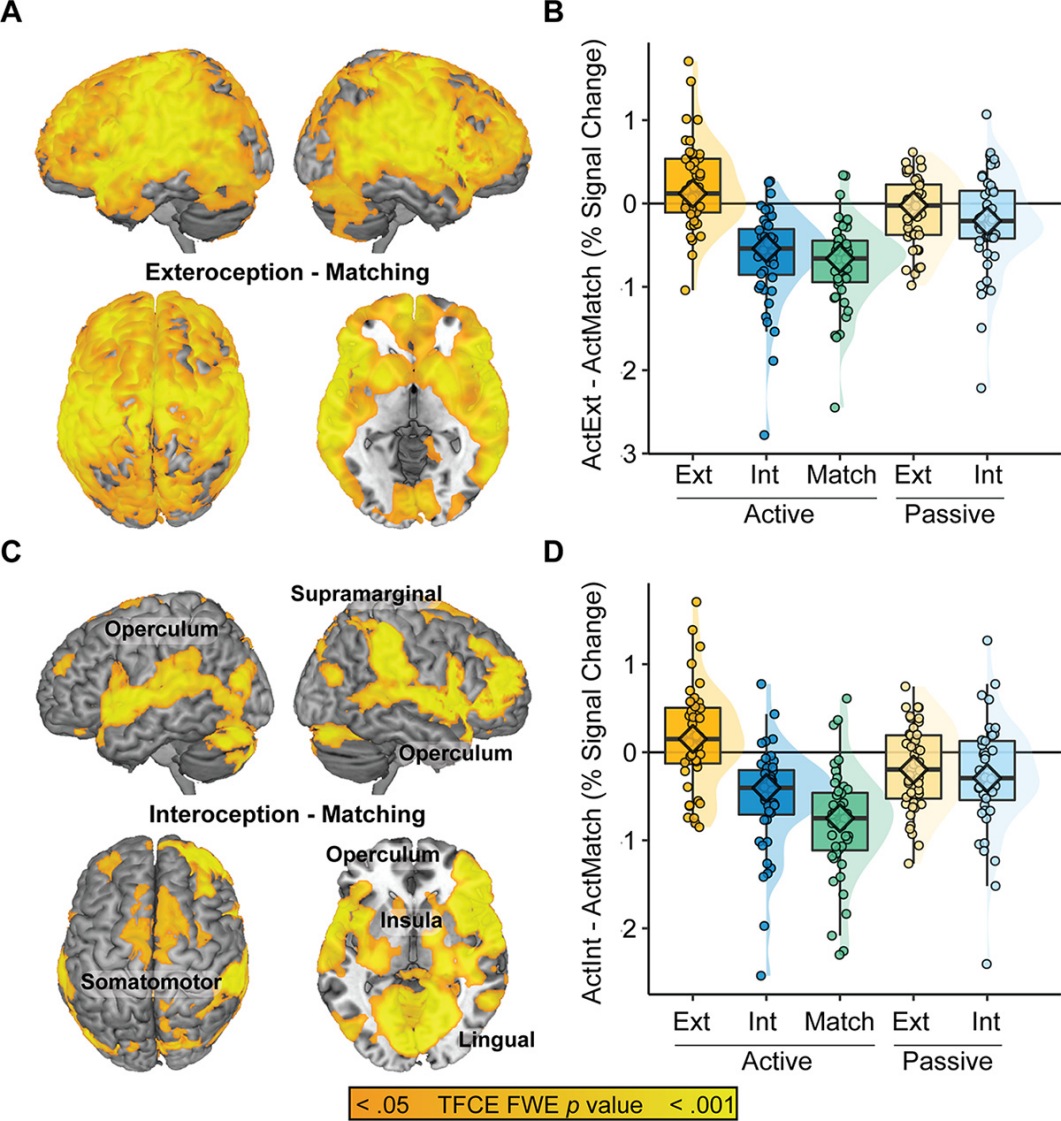
Endogenous versus exogenous sources of interoceptive control. ***A***, Active Exteroception > Active Matching replicated the deactivation effect observed for Active Interoception. ***B***, Median % signal change across the regions identified via the [Active Exteroception > Active Matching] contrast. ***C***, Active Interoception > Active Matching linked endogenous respiration to greater activation along an insula-operculum pathway relative to the exogenous control condition. ***D***, Median % signal change across the regions identified via the [Active Interoception > Active Matching] contrast. Ext = Exteroception; Int = Interoception.

The next planned comparison contrasted Active Matching against Active Interoception to explore endogenous and exogenous sources of respiratory control. To contextualize Active Matching, we first compared it to Active Exteroception. Active Matching showed even more pronounced and widespread patterns of deactivation than Active Interoception (Fig. 3*C* vs Fig. 5*A*). Compared with the endogenously paced Active Interoception condition, the exogenously-paced Active Matching condition led to greater deactivation bilaterally along an insula/operculum pathway, somatomotor regions, and within the ventral occipital cortex and cerebellum (Fig. 5; Table 10).

### Psychophysiological interaction (PPI) analysis

**Figure 6.**
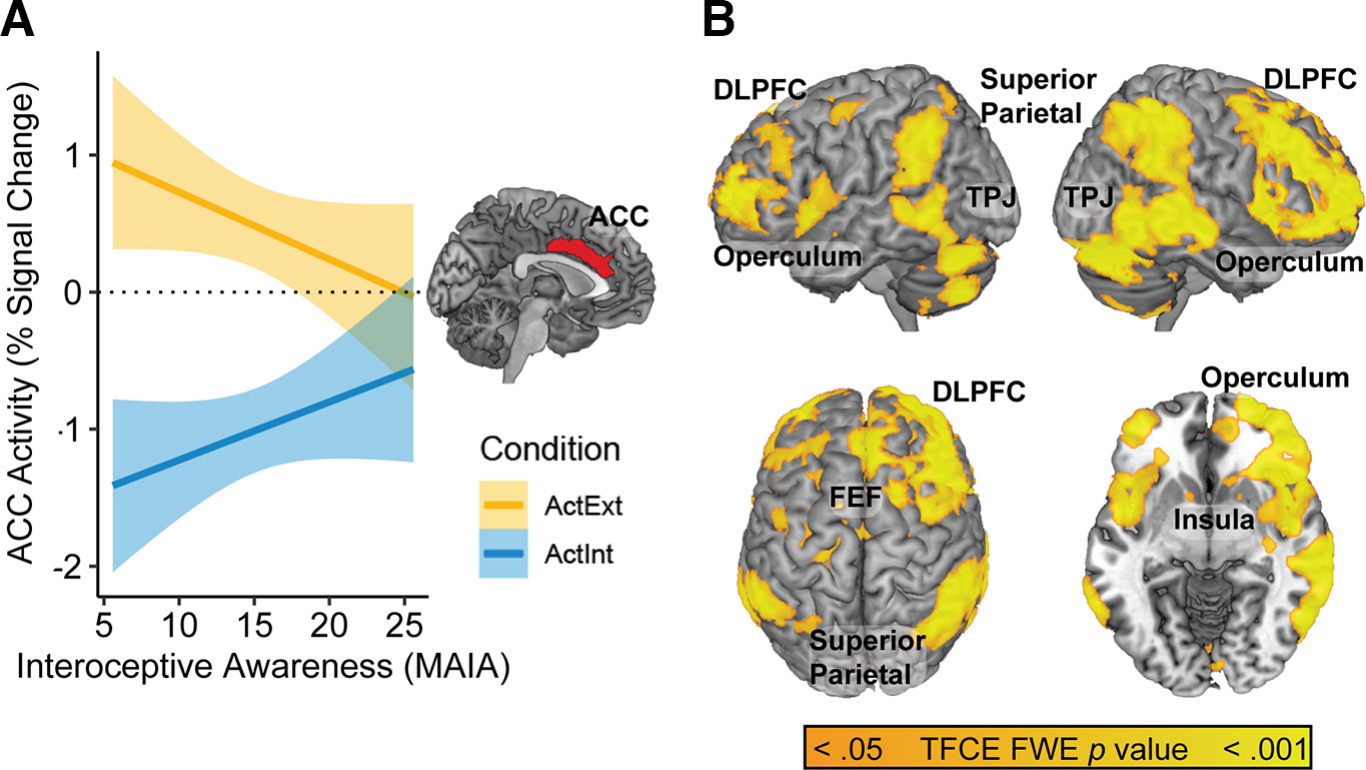
Psychophysiological interaction (PPI) analysis. ***A***, The ACC seed region obtained from the MAIA covariate analysis. ***B***, PPI between ACC regional activity and task conditions [Active Interoception > Active Exteroception], demonstrating enhanced ACC connectivity with a frontoparietal network during Active Interoception relative to Active Exteroception. ACC = Anterior Cingulate Cortex; DLPFC = Dorsolateral Prefrontal Cortex; TPJ = Temporoparietal Junction; ActExt = Active Exteroception; ActInt = Active Interoception.

**Figure 7.**
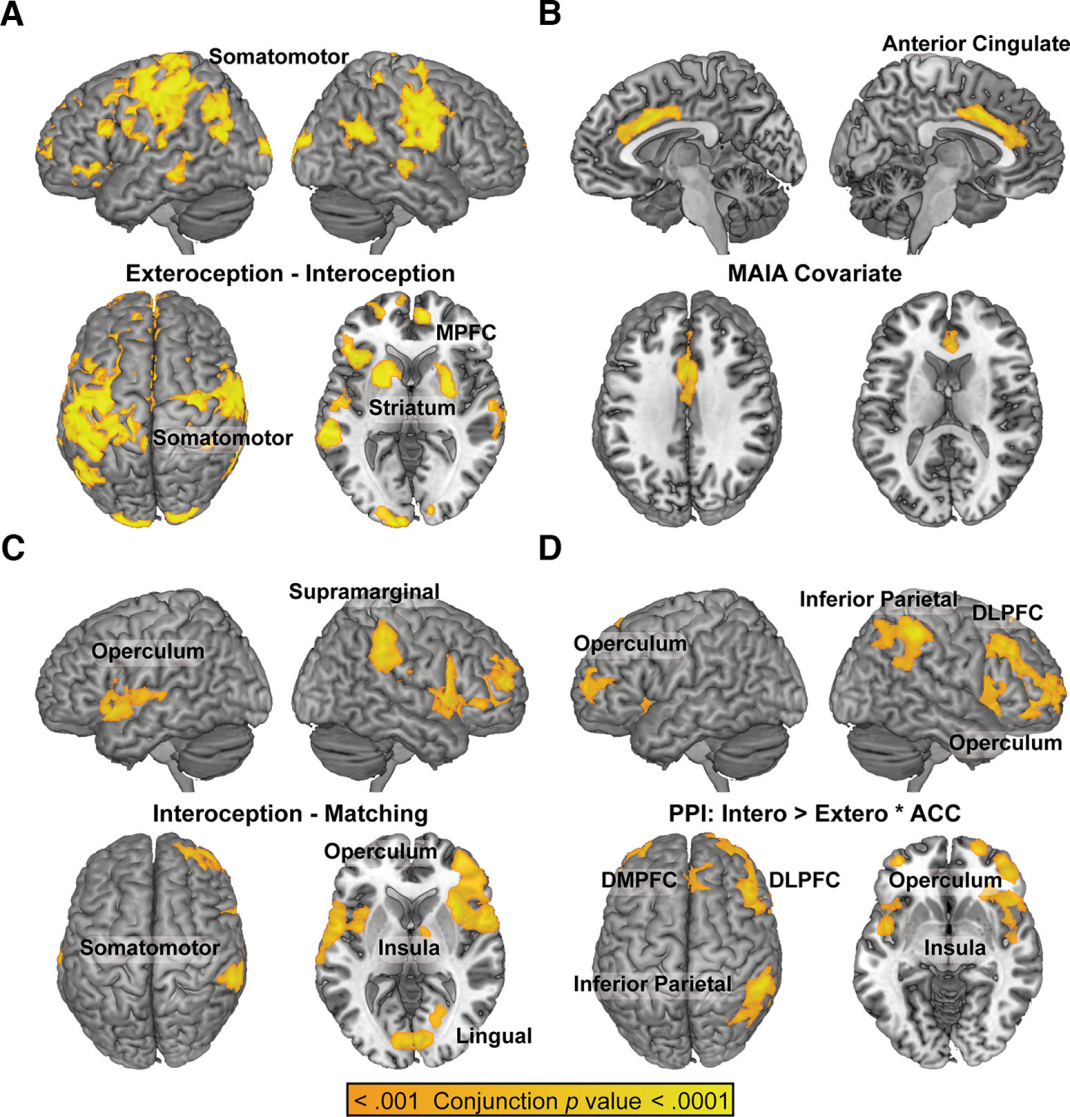
Test-retest reliability of neuroimaging findings. To demonstrate the robustness and replicability of the results, conjunction analysis for the four main contrasts reported in this paper was conducted. As the TFCE algorithm does not currently perform conjunction analysis, and halving the sample size reduces experimental power, the baseline and postintervention sessions were each analyzed separately at *p* < 0.001^1/2^ = *p* < 0.0316, so that the resulting overlap would yield a conjunction *p* < 0.001; a cluster size of k ≥ 500 was also applied to each estimate as this cluster size resulted in a FWE corrected *p* value < 0.05 for *p* < 0.001. ***A***, the contrast of Active Exteroception – Active Interoception reproduced the pattern of somatomotor and prefrontal activation. ***B***, The MAIA covariate of [Active Exteroception – Active Interoception] reproduced the anterior cingulate region of interest. ***C***, The contrast of Active Interoception – Active Matching reproduced greater insula/opercular activation for endogenously-paced interoception (Active Interoception) than exogenously-paced interoception (Active Matching). ***D***, The PPI analysis reproduced increased connectivity between the anterior cingulate and the dorsal attention network (DAN). DMPFC = dorsomedial prefrontal cortex; DLPFC = dorsolateral prefrontal cortex; ACC = anterior cingulate.

The final contrast examined changes in functional connectivity between Active Exteroception and Active Interoception. The anterior cingulate cortex (ACC) region of interest (Figs. 4*A*, 6*A*) was entered as a seed region in a psychophysiological interaction (PPI) analysis. The PPI analysis explored changes in functional connectivity with the ACC (Fig. 7*C*) as a function of the two experimental conditions (Active Interoception vs Active Exteroception). The analysis revealed a strong integration of the ACC into regions consistent with the DAN during Active Interoception (Fig. 6*B*; Table 11).

**Table 10:** Comparisons between active effects and active match

		Cluster	Peak *p*	Peak	Peak	Peak *p*	Coordinates (MNI)		
Description	Side	size (k)	(FWE)	TFCE	Z	(raw)	*x*	*y*	*Z*
ActExt > Act Match									
Cerebral cortex	-	134,079	0	6116.73	3.54	<0.001	−58	−24	16
ActInt > Act Match									
Insula, operculum, ventral striatum, lingual gyrus	R	54,630	0	3898.16	3.54	<0.001	60	10	−2
Dorsolateral PFC	L	706	0.011	1435.25	3.54	<0.001	−32	54	26
Middle temporal gyrus	L	10	0.049	952.43	3.54	<0.001	−52	−42	−6

ActExt = Active Exteroception; ActInt = Active Interoception; ActMatch = Active Matching.

**Table 11:** PPI effects

		Cluster	Peak *p*	Peak	Peak	Peak *p*	Coordinates (MNI)		
Description	Side	size (k)	(FWE)	TFCE	Z	(raw)	*x*	Side	Size (k)
Dorsal attention network: frontal	R	23783	<0.001	3269.41	3.54	0	44	24	40
Dorsal attention network: parietal	R	18671	0.001	2368.12	3.54	0	58	−40	52

The PPI was defined by the interaction of the [ActInt – ActExt] contrast * ACC activity.

### Test-retest reliability

A conjunction analysis of the main H1–H4 contrasts estimated separately at each of the two timepoints successfully reproduced the results described above (Fig. 7).

## Discussion

Relative to visual Exteroception, respiratory Interoception was characterized by pervasive neural deactivation in prefrontal, somatomotor, striatum, and temporoparietal regions. This finding deviates from previous respiratory interoception studies (Farb et al., 2013b; Wang et al., 2019), where more difficult sensory tasks yielded greater DAN activity relative to greater DMN activity for less difficult tasks. The finding also differs from liminal heartbeat detection paradigms (cf. Ring and Brener, 2018), where prior research has related interoceptive accuracy to greater activity and connectivity within the SLN (Critchley et al., 2004; J.S.X. Chong et al., 2017; Tan et al., 2018). The lack of SLN involvement in closely matched, supraliminal tasks supports recent arguments that SLN engagement generally indicates perceptual decision-making rather than interoception specifically (Koeppel et al., 2020; Baltazar et al., 2021). Why attending to the breath reduces brain activity without impairing task performance remains an intriguing question. Performance may be sustained because of noise inhibition along existing representational pathways (cf. Kuehn et al., 2016), which could offset the disadvantage seemingly implied by widespread cortical deactivation.

Alternatively, slower and deeper breathing during the Active Interoception condition could suggest that neural activation differences may be predicated on gross physiological changes driven by different respiration rates, RVT, and spontaneous end-tidal CO_2_ (Petco_2_; Golestani et al., 2015). Modelling RVT successfully controlled for some pervasive deactivation effects (Extended Data Fig. 2-1), yet controlling for respiration rate and RVT at both the first (acquisition volume) and second (task block) levels of analysis failed to eliminate the Interoception deactivation effect. Furthermore, the rate of respiratory slowing (∼0.02 Hz) was too small to provoke hypocapnia, and while CO_2_ was not measured in this study, convergent findings suggest that controlling for Petco_2_ reduces functional connectivity but not task related activity (Madjar et al., 2012; Golestani and Chen, 2020). Finally, the Active Matching task, in which respiration frequency and yoking of behavior were more closely matched to Active Exteroception, still resulted in this deactivation pattern. While there must certainly be interactions between attention and physiology driving the deactivation effect, they seem unlikely to be explained by gross changes in blood oxygenation or CO_2_.

The right anterior insula, commonly implicated in investigations of interoceptive accuracy (Critchley et al., 2004; Wang et al., 2019; Harrison et al., 2021; Haruki and Ogawa, 2021), was insensitive to contrasts between actively-reported Interoception and Exteroception. However, the insula was implicated in contrasts between Active Interoception and Active Matching, where moving from endogenous to exogenous respiratory pacing resulted in greater insula deactivation. This effect is not simply because of the integration of external cues, as this deactivation effect was absent in Active Exteroception. Endogenous respiratory rhythms may be continuously processed by this insula/operculum pathway, revealed only when such processing is disrupted by task demands. Here, exogenous control over the respiratory cycle may have disrupted sensory integration or signals such as the body’s internal respiratory “clock,” a rhythm commonly attributed to the pre-Bötzinger complex (J.C. Smith et al., 1991), but which may communicate with the insula to support homeostasis (Yang and Feldman, 2018). By this logic, liminal detection accuracy paradigms implicate the insula precisely because they are comparing participants with robust heartbeat representations to those where such discrimination is compromised, disrupted, or absent. Conversely, attention to salient signals such as the respiratory rhythm seem unlikely to implicate the anterior insula unless compared with situations where breath awareness is somehow disrupted or compromised.

### The moderating influence of self-reported interoceptive awareness

A second aim of the study was to explore the potential moderating effects of subjective interoceptive awareness (MAIA scores) on interoceptive network engagement, with the hypothesis that greater subjective interoceptive awareness would be supported by increased SLN activity in the ACC and anterior insula, commensurate with their established role in supporting interoceptive accuracy (Critchley et al., 2004; J.S.X. Chong et al., 2017; Harrison, Köchli, et al., 2021).

Despite the surprising widespread pattern of cortical deactivation during interoception of the breath, the ACC was spared from deactivation in participants who generally reported greater interoceptive awareness via the MAIA total score. Exploratory analyses of the MAIA subfactors suggested that this moderating effect was driven primarily by the Emotion Awareness, Self-Regulation, and Body Listening subfactors, which were intended to assess “Mind-Body Integration” during the scale’s construction (Mehling et al., 2022). Furthermore, Attention Regulation implicated the ACC but not left-lateralized language regions, while the converse was true for Noticing. Preserved activity in brain regions supporting attention regulation (ACC) and the articulation of experience (language regions) together appear to support reports of interoceptive integration.

### Characterizing the network supporting interoceptive awareness

The final aim of the study was to characterize an interoceptive attention network supporting adaptive interoceptive sensibility. The rostral ACC was identified as a seed region for psychophysiological interaction (PPI) analysis, convolving the activity in the region implicated as a MAIA covariate with the Active Interoception versus Active Exteroception contrast.

Breath interoception led to increased connectivity between the ACC and the frontoparietal dorsal attention network (DAN; Spreng et al., 2013; Szczepanski et al., 2013). While DAN activity was inhibited during interoception, those with greater self-reported interoceptive awareness were likely to offset such deactivation by greater connectivity between the ACC and DAN, a potential biomarker of interoceptive engagement. This finding helps to explain how interoceptive tracking accuracy was equivalent to exteroceptive tracking accuracy despite reduced cortical activity. Interoceptive processing may be continuous and automatic, but largely obscured by a combination of external sensory signals and internal cognitive processing. Interoceptive attention may therefore employ “addition by subtraction,” a reduction of competing neural representations rather than the activation of a dormant interoceptive pathway.

The idea of an “always on” interoceptive state is consistent with contemporary theories that place interoception as the background of consciousness, such as Damasio’s “Proto Self” (Bosse et al., 2008) or “Core Consciousness” (Parvizi and Damasio 2001), and the distinction between a subcortical “Core Self” and higher order representations in the cerebral cortex (Northoff and Panksepp, 2008). Interoception is likely the first sense represented in developing brains because of the critical roles homeostasis plays in ensuring survival (Fotopoulou and Tsakiris, 2017; Filippetti, 2021). At the attentional level, the consistent presence of periodic interoceptive signals may lead to neural habituation, in keeping with neural repetition suppression effects for familiar and repeatedly represented sensory signals (Summerfield et al., 2008; Barron et al., 2016). Relative to the cortical activation observed when attending to the exteroceptive senses (Liu et al., 2005; Alho et al., 2014), interoception of salient signals such as the breathing cycle may require overcoming the “learned irrelevance” of historically ignored sensory information (Segaert et al., 2013).

### Limitations and constraints on generalizability

The IEAT should be improved by introducing a circle motion to the Passive Interoception condition, as the stationary target described here led to a motion confound between passive interoception and the other four experimental conditions. In its current form, Passive Interoception showed relative deactivation in the middle temporal (MT or V5) region of the visual cortex, which is well-established for its sensitivity to motion (Albright and Stoner, 1995). Future iterations of the task might therefore include regular visual stimulus motion in the passive interoception condition to allow parity in motion across all task conditions.

Additionally, while we corrected for both respiration frequency and RVT, we did not measure Petco_2_, which requires the use of nasal cannula (Chang and Glover, 2009) or a facemask for CO_2_ capnography (Golestani and Chen, 2020). While we do not attribute the findings to CO_2_ for reasons described above, the possibility remains that the interoceptive conditions evoked unexpected changes to CO_2_ that were unrelated to either of frequency or RVT. Including Petco_2_ measurements would help to rule out this confounding explanation for the cortical deactivation effects.

Several constraints on generalizability are also apparent. We present unexpected and exploratory findings from a small sample; replication is therefore needed, especially given the paucity of studies in this area. The community sample may also not generalize to people with clinical conditions associated with interoceptive dysfunction, nor advanced contemplative practitioners with extensive interoceptive training. Respiration is also only one of many interoceptive signals that each vary in their reportability and controllability, and so may possess distinct neural dynamics. Multimodal interoceptive research is needed to determine the generalizability of the patterns discussed here, to clarify the impact of active reporting versus passive engagement, the role sensory signal salience, and the influence of participant expertise/dysfunction.

A further limitation to the present findings is that all practices were conducted with eyes-open, which may be dissimilar to many meditation practices. Following repeated evidence that opening one’s eyes decreases the functional connectivity between the SLN and the DMN, it was theorized that reducing the connectivity between these networks reflects an orientation to external rather than internal events (Han et al., 2020). However, here interoceptive attention with eyes open led to greater connectivity between the ACC seed region (an efferent hub of the SLN) and both the DAN and more posterior aspects of the insula rather than the DMN. It remains possible that dynamics for interoceptive awareness of the breath may differ in eyes-closed paradigms. Increased DMN coupling may also represent a distinct form of internal awareness at the elaborative, semantic level of processing distinct from interoceptive awareness, as has been previously proposed (Gusnard et al., 2001; Andrews-Hanna et al., 2014).

In conclusion, relative to Exteroception, Interoception of the breath reduced somatomotor and prefrontal activity, offset by enhanced connectivity between the ACC and the DAN. Greater self-reported interoceptive awareness was linked to sparing of the ACC and language processing regions from this deactivation pattern. Together, these processes may allow sensory signals from the body to be better discerned. The ACC deactivation observed in the IEAT paradigm may serve as a candidate biomarker of individual differences in interoceptive processing, one that distinguishes adaptive representations engendered by contemplative training from the avoidance and catastrophizing observed in anxiety and somatic disorders. Larger, more diverse samples and replication of effects are needed to test these emerging ideas.

## Synthesis

Reviewing Editor: Erica Glasper, The Ohio State University Decisions are customarily a result of the Reviewing Editor and the peer reviewers coming together and discussing their recommendations until a consensus is reached. When revisions are invited, a fact-based synthesis statement explaining their decision and outlining what is needed to prepare a revision will be listed below. The following reviewer(s) agreed to reveal their identity: NONE. Note: If this manuscript was transferred from JNeurosci and a decision was made to accept the manuscript without peer review, a brief statement to this effect will instead be what is listed below.

Overview: The authors of this manuscript describe a new fMRI task that utilizes respiratory interoceptive attention, with the goal of using it to probe the neural basis of interoception in studies that could follow. The authors selected a small sample of health individuals from a larger clinical trial using a mindfulness intervention that focused on body awareness. These individuals reported higher levels of stress during this task. The authors report that, despite their initial predictions, participants demonstrated widespread deactivations, rather than activations, of the insular cortex and other brain regions. The task is sound and the study, overall, is meritorious. There are a few minor concerns, that require further attention that are described, in detail, below. When needed, exact reviewer comments can be found in quotations.

Overall Comments

The authors are encouraged to reduce, or remove, the many leading statements within the manuscript. Please see the following as examples and consider phrasing that mentions an association between 2 variables:

1. Abstract - ‘attention towards...may lead to a low activity, high connectivity state’

2. Discussion - ‘Interoception led to increased connectivity in the frontoparietal dorsal attention network’

Remaining Concerns

Statistics

1. It is unclear why the authors used a global MAIA score instead of conducting analyses on the validates subscales. If a multiple comparison issue is of concern, this analysis could be included in the supplemental material to highlight “whether the observed brain-behavior relationships could be further refined”.

2. 22 participants in a small N size, and not a ‘relatively small’ N like the author’s state. This should be explicitly emphasized in the revised manuscript and the authors should state that replication is needed given this small N.

Discussion

1. It is suggested that the authors reconsider the “notion of MAIA scores simply as a measure of ‘interoceptive sensibility’ in the discussion”. The authors should focus on the most relevant subscale processes.

2. It is suggested that the authors reduce, or narrow, the respiratory interoceptive network inferences. The full extent to which these findings may apply to other interoceptive sensations was not studied.

## Author Response

Dear Professor Glasper,

Thank you for your efforts in reviewing our manuscript entitled “Interoceptive Awareness of the Breath Preserves Dorsal Attention Network Activity amidst Widespread Cortical Deactivation: A Within-Participant Neuroimaging Study“, submitted for consideration in eNeuro.

We appreciate your supportive synthesis of the paper, reviews, and response to reviews. We are grateful to the reviewers for their suggestions, which we believe have helped us to substantially improve the paper. We respond to each of the comments below.

Overall Comments. The authors are encouraged to reduce, or remove, the many leading statements within the manuscript. Please see the following as examples and consider phrasing that mentions an association between 2 variables:

1. Abstract - ‘attention towards...may lead to a low activity, high connectivity state’

We have replaced this general statement with more specific language: “attention towards accessible body sensations such as the breath may lead to greater ACC-Dorsal Attention Network connectivity state in a context of widespread cortical deactivation.”

2. Discussion - ‘Interoception led to increased connectivity in the frontoparietal dorsal attention network’

We clarify that “Breath interoception led to increased connectivity between the ACC and the frontoparietal dorsal attention network”.

Remaining Concerns

Statistics

1. It is unclear why the authors used a global MAIA score instead of conducting analyses on the validates subscales. If a multiple comparison issue is of concern, this analysis could be included in the supplemental material to highlight “whether the observed brain-behavior relationships could be further refined”.

We appreciate the suggestion to delve deeper than a blanket ‘sensibility’ description of the MAIA, and the reviewer is correct that we shouldn’t equivocate- we have revised the description of MAIA to be an assessment of subjective interoceptive awareness and attention tendency throughout the manuscript.

We also agree that greater specificity of our findings could be supported by reviewing the interoceptive subscales. We initially created a second supplement to the paper (“Supplementary MAIA Analyses”), but learned that eNeuro does not allow supplemental materials, so we have placed extensive analyses within a document on the Open Science Framework for your reference (https://osf.io/d3uzw), with the highlights of these analyses added to the manuscript text.

The supplement reviews the creation of the MAIA and its conceptual and empirical subfactors, and explains why we chose to use the single total score given limitations with the original version of the MAIA that was available at the time of data acquisition. We support this decision via exploratory factor analysis of the observed data, showing an optimal single-factor solution, and also that many of the subfactors show similar correlations with anterior cingulate cortex (ACC) region of interest (ROI).

We did take to heart the need to go deeper than a blanket total score; we therefore explored each of the eight MAIA subfactors at both the TFCE familywise-error corrected and a more liberal threshold (p<.005, k{greater than or equal to}200), we now attach figures of these analyses as extended data to the MAIA analysis figure (Figure 4-1, Figure 4-2, and Figure 4-3). We also now briefly discuss these results in the revised results and methods section of the manuscript.

The reviewer was correct that these analyses show more directly how whole-brain covariates might be supported by the subscales. The MAIA total score covariate map is similar to reduced- threshold maps for the subfactors of Emotion Awareness, Self-Regulation, Body Listening, and to a lesser extant Attention Regulation and Noticing. These whole brain analyses suggest a particular role of the “Mind-Body Integration” conceptual domain in supporting the covariation effect. Intriguingly, Attention Regulation implicated only the ACC component of the total score map, whereas Noticing implicated only language regions. By contrast the Not Distracting, Not Worrying, Trusting, and Noticing subfactors show little of this covariation effect, suggesting that self-reported integration/use of interoceptive information may be driving the total score finding. We make note of these ideas in the revised discussion section.

2. 22 participants in a small N size, and not a ‘relatively small’ N like the author’s state. This should be explicitly emphasized in the revised manuscript and the authors should state that replication is needed given this small N.

We appreciate the need to be clear about small sample size and have removed the word ‘relatively’ from the sentence, which now reads “We present unexpected and exploratory findings from a small sample; replication is therefore needed, especially given the paucity of studies in this area.” (p.32)

Discussion

1. It is suggested that the authors reconsider the “notion of MAIA scores simply as a measure of ‘interoceptive sensibility’ in the discussion”. The authors should focus on the most relevant subscale processes.

We agree that the subscales add nuance, and also that ‘sensibility’ may not be the appropriate term for what the MAIA measures- we have revised the manuscript to emphasize ‘attention tendency’ rather than ‘sensibility’ per se, which seems to be a narrower term focusing on appraisal. As noted above, the suggested subscale analyses provide supporting evidence that these effects are driven primarily by the integration subscales (Emotion Awareness, Self-Regulation, and Body Listening), and we now dedicate a paragraph of the results and discussion to reviewing these findings, in addition to a more lengthy discussion of these findings in the MAIA Analysis Supplement found on the Open Science Framework (https://osf.io/d3uzw) as eNeuro will not allow supplemental materials beyond additional tables and figures.

2. It is suggested that the authors reduce, or narrow, the respiratory interoceptive network inferences. The full extent to which these findings may apply to other interoceptive sensations was not studied.

This suggestion is well-received given the multimodal nature of interoception. We have revised the manuscript language to be more specific about respiratory interoception / attention to the breath rather than implying that the findings generalize to interoception more broadly.

We are grateful for the opportunity to improve our work and hope that the revisions satisfy the constructive suggestions offered by the editor and reviewers.

Thank you for time and consideration. We look forward to your feedback!

Best regards,
