# Supplementary figures and images for: Interoceptive Awareness of the Breath Preserves Attention and Language Networks amidst Widespread Cortical Deactivation: A Within-Participant Neuroimaging Study
Source: eNeuro. 2023 Jun 23;10(6):ENEURO.0088-23.2023. doi: 10.1523/ENEURO.0088-23.2023 (PMC10295813; doi:10.1523/ENEURO.0088-23.2023)

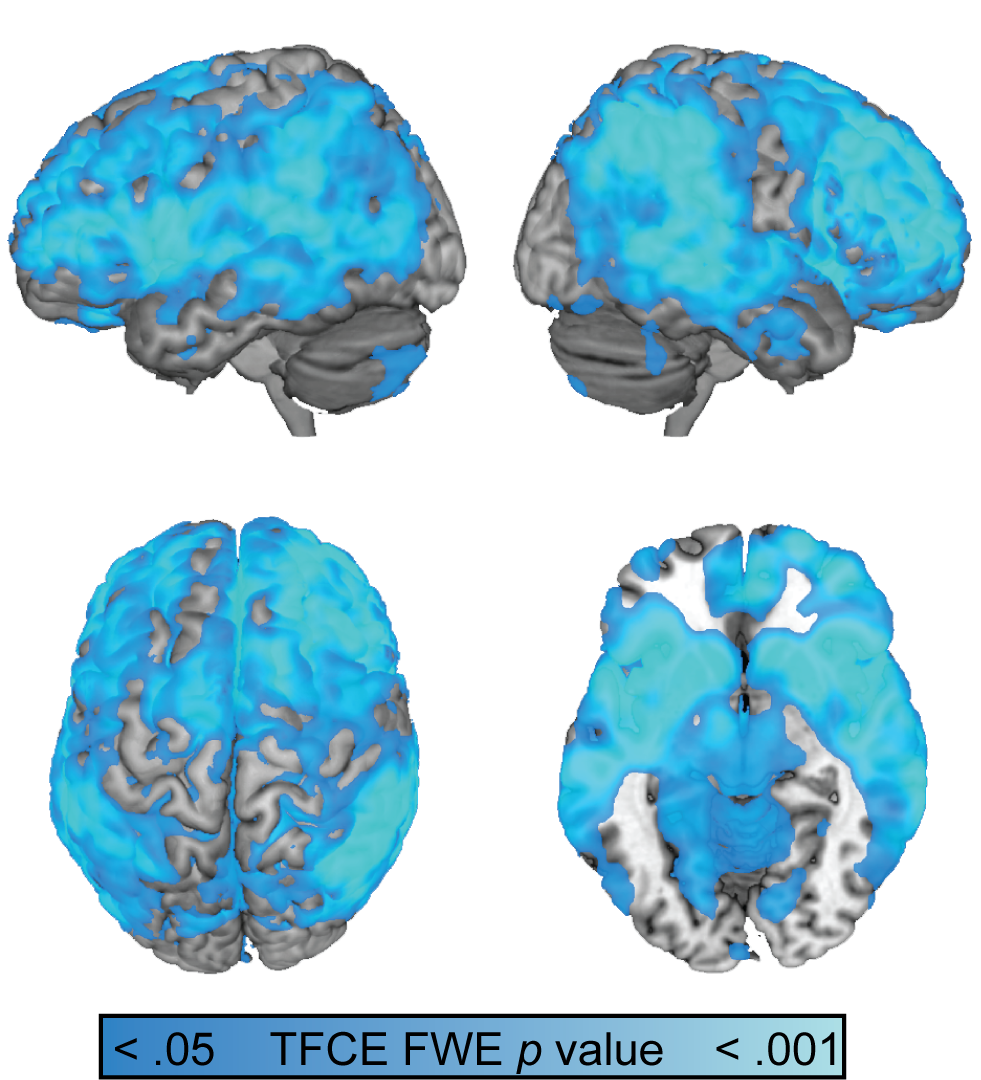

Supplement: Extended Data Figure 2-1 — Effects of the respiratory volume/time (RVT) covariate on BOLD activity. Contrasts maps were generated from the five-condition model corrected using threshold-free cluster estimation (TFCE) to the familywise p < 0.05 level. Areas in blue demonstrate reduced BOLD activity with greater levels of RVT. Download Figure 2-1, TIF file. [file enu-eN-NWR-0088-23-s01.tif]

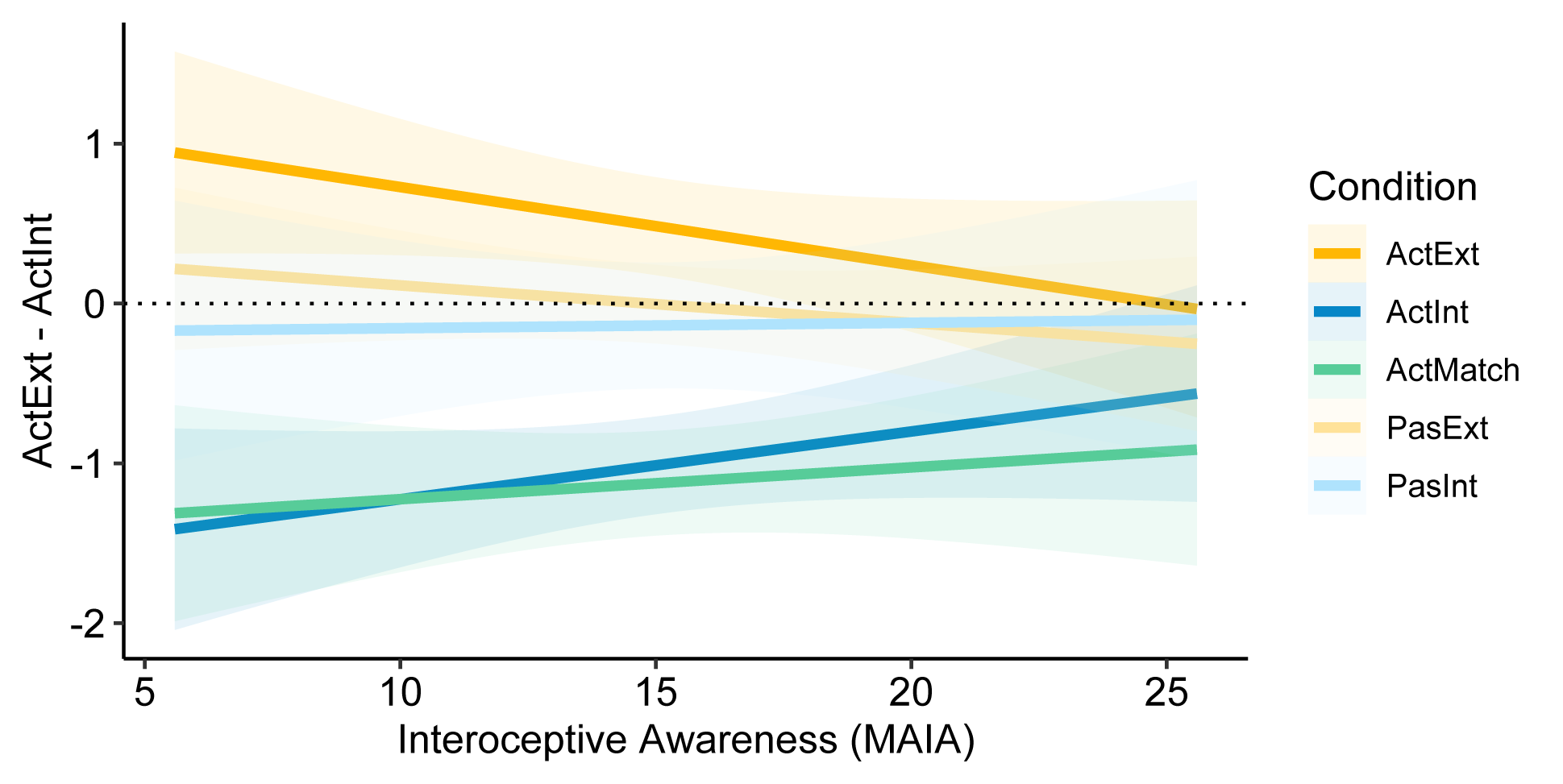

Supplement: Extended Data Figure 4-1 — MAIA scale and anterior cingulate (ACC) activation with all five task conditions. The relationship between interoceptive sensibility/awareness scores (MAIA) and anterior cingulate activity across all five task conditions. Signal was extracted from each experimental condition and plotted above, demonstrating the same reduced separation between conditions, including within the passive conditions. ActExt = Active Exteroception, ActInt = Active Interoception, ActMatch = Active Matching, PasExt = Passive Exteroception, PasInt = Passive Interoception. Download Figure 4-1, TIF file. [file enu-eN-NWR-0088-23-s02.tif]

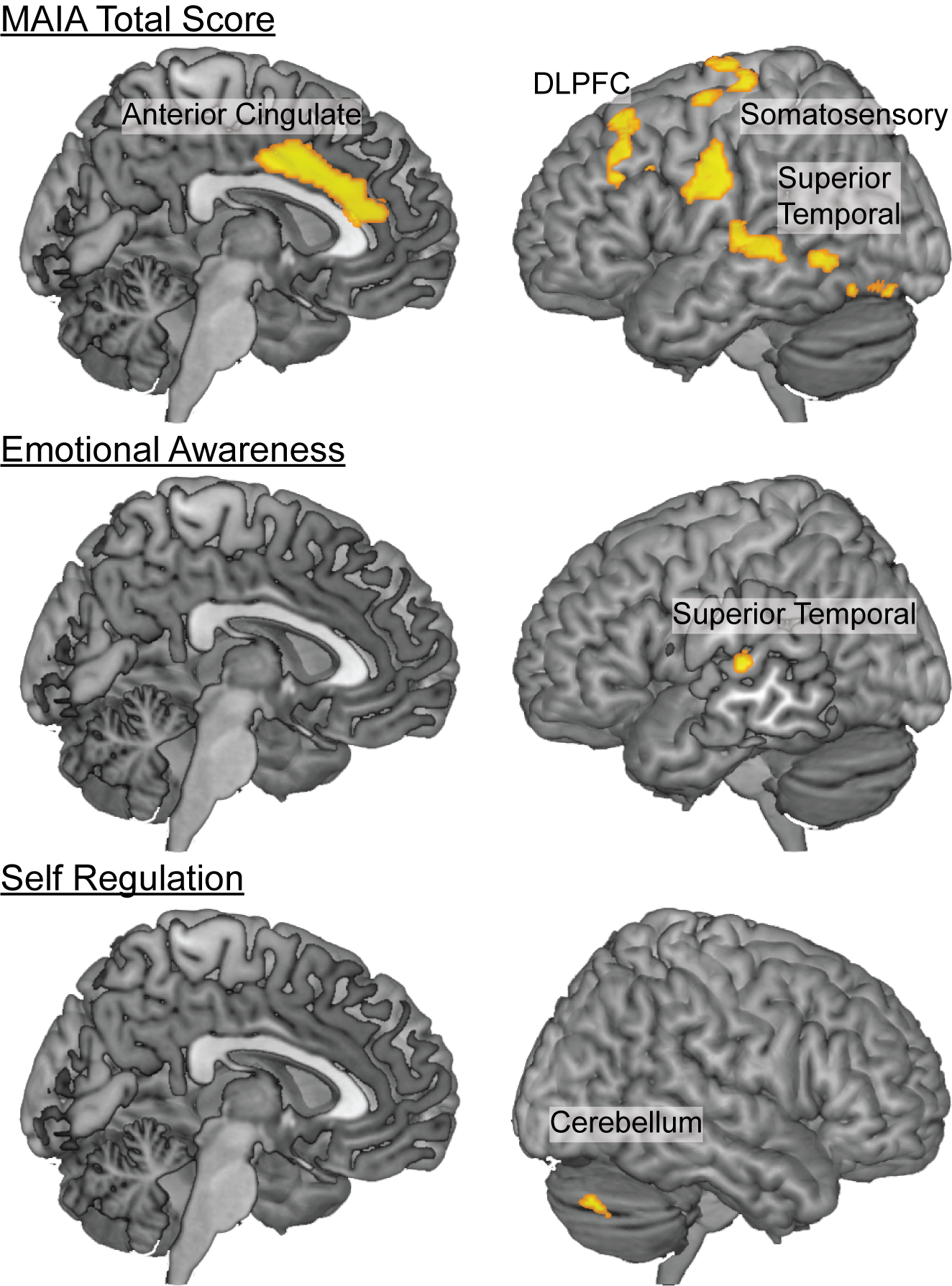

Supplement: Extended Data Figure 4-2 — MAIA covariates of the [Active Interoception – Active Exteroception] contrast, corrected using threshold-free cluster estimation (TFCE) to the familywise p < 0.05 level. The analysis was run on each MAIA subscale in place of the total score as a covariate in a model that also controlled for study design (Group × Time), respiration frequency, and RVT. Only covariates with significant voxels are displayed; only Emotional Awareness and Self-Regulation showed evidence of covariation. Emotional Awareness also showed a TFCE-corrected cluster of activity in the left planum temporal/superior temporal gyrus. Self-Regulation also showed a TFCE-corrected cluster of activity in the right lateral cerebellum at the border of Crus1 and Crus2. Download Figure 4-2, TIF file. [file enu-eN-NWR-0088-23-s03.tif]

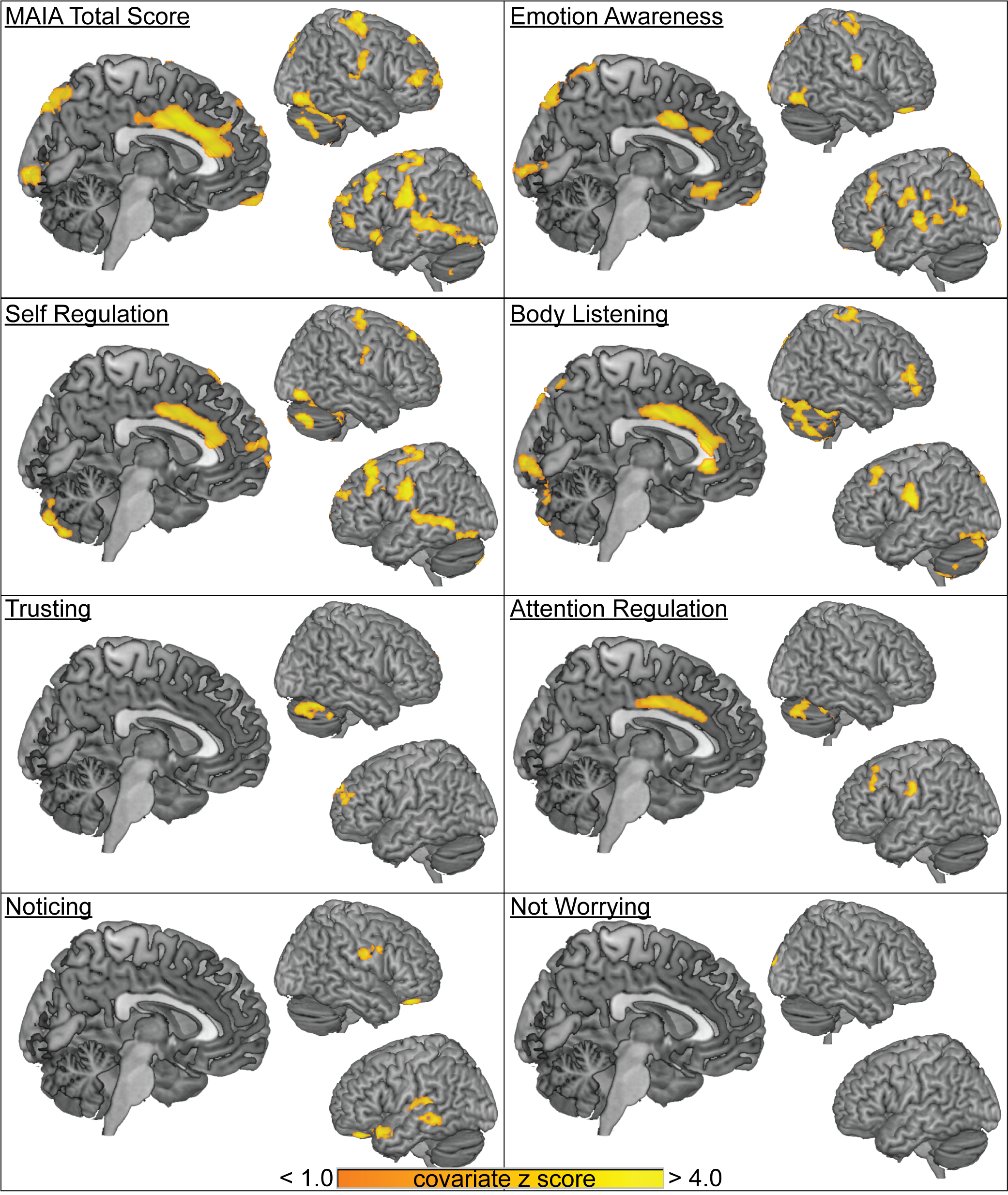

Supplement: Extended Data Figure 4-3 — MAIA covariates of the [Active Interoception – Active Exteroception] contrast, corrected using the joint thresholds of p < .005 and cluster size k ≥ 200 voxels. The analysis was run on each each MAIA subscale in place of the Total score as a covariate in a model that also controlled for study design (Group x Time), respiration frequency, and RVT. Only covariates with significant voxels are displayed. As illustrated above, many components shared substantial overlap with the pattern observed in the MAIA total score. Emotion Awareness, Self-Regulation, Body Listening, and to a lesser extent Attention Regulation and Noticing all showed similar patterns of covariation, a pattern that aligns with the ACC ROI correlations described in Table 4-3. These whole brain analyses do suggest that Emotion Awareness, Self-Regulation, and Body Listening also implicate the left lateralized language regions, suggesting a particular role of the “Mind-Body Integration” conceptual domain in supporting the covariation effect. Noticing only implicated the temporal and somatosensory association aspects of the total score covariate, and conversely, Attention Regulation implicated the ACC without many of the left-temporal language regions. There was little overlap with Trusting, which only implicated the left frontal pole and right cerebellum (Crus 1), Not Worrying, which only implicated the right superior occipital cortex, and no covariation with Not Distracting even at this exploratory threshold. Download Figure 4-3, TIF file. [file enu-eN-NWR-0088-23-s04.tif]
