# Supplementary material for: Interoceptive Awareness of the Breath Preserves Attention and Language Networks amidst Widespread Cortical Deactivation: A Within-Participant Neuroimaging Study
Source: eNeuro. 2023 Jun 23;10(6):ENEURO.0088-23.2023. doi: 10.1523/ENEURO.0088-23.2023 (PMC10295813; doi:10.1523/ENEURO.0088-23.2023)
Supplement: Extended Data Table 4-1 — MAIA Scale subfactor intercorrelations and reliabilities. Download Table 4-1, DOCX file. [file enu-eN-NWR-0088-23-s05.docx]

| **Table 4-1.** MAIA Scale subfactor intercorrelations and reliabilities. | | | | | | | | | |
| --- | --- | --- | --- | --- | --- | --- | --- | --- | --- |
|  | **1** | **2** | **3** | **4** | **5** | **6** | **7** | **8** | **9** |
| 1. Total | **0.85** |  |  |  |  |  |  |  |  |
| 2. Noticing | 0.68 | **0.76** |  |  |  |  |  |  |  |
| 3. Not Distracting | 0.21 | 0.18 | **0.75** |  |  |  |  |  |  |
| 4. Not Worrying | 0.49 | 0.21 | 0.09 | **0.77** |  |  |  |  |  |
| 5. Attention Regulation | 0.92 | 0.55 | 0.23 | 0.49 | **0.92** |  |  |  |  |
| 6. Emotion Awareness | 0.67 | 0.69 | 0.1 | 0.09 | 0.58 | **0.80** |  |  |  |
| 7. Self-Regulation | 0.94 | 0.63 | 0.17 | 0.52 | 0.84 | 0.65 | **0.82** |  |  |
| 8. Body Listening | 0.85 | 0.56 | 0.31 | 0.25 | 0.82 | 0.54 | 0.79 | **0.89** |  |
| 9. Trusting | 0.51 | 0.27 | -0.17 | 0.34 | 0.51 | 0.31 | 0.52 | 0.45 | **0.87** |
