# Supplementary material for: Interoceptive Awareness of the Breath Preserves Attention and Language Networks amidst Widespread Cortical Deactivation: A Within-Participant Neuroimaging Study
Source: eNeuro. 2023 Jun 23;10(6):ENEURO.0088-23.2023. doi: 10.1523/ENEURO.0088-23.2023 (PMC10295813; doi:10.1523/ENEURO.0088-23.2023)
Supplement: Extended Data Table 4-2 — Factor loadings for a single factor solution of the eight MAIA subfactors. Download Table 4-2, DOCX file. [file enu-eN-NWR-0088-23-s06.docx]

| **Table 4-2.** Factor loadings for a single factor solution of the eight MAIA subfactors. | | |
| --- | --- | --- |
| **Subfactor** | **Loading** |  |
| Noticing | 0.68 |  |
| Attention Regulation | 0.92 |  |
| Emotion Awareness | 0.67 |  |
| Self-Regulation | 0.96 |  |
| Body Listening | 0.84 |  |
| Trusting | 0.52 |  |
| Not Distracting | 0.20 |  |
| Not Worrying | 0.42 |  |
