# Supplementary material for: Interoceptive Awareness of the Breath Preserves Attention and Language Networks amidst Widespread Cortical Deactivation: A Within-Participant Neuroimaging Study
Source: eNeuro. 2023 Jun 23;10(6):ENEURO.0088-23.2023. doi: 10.1523/ENEURO.0088-23.2023 (PMC10295813; doi:10.1523/ENEURO.0088-23.2023)
Supplement: Extended Data Table 4-3 — Univariate Correlations of Anterior Cingulate ROI with MAIA total score and subscales. Download Table 4-3, DOCX file. [file enu-eN-NWR-0088-23-s07.docx]

| **Table 4-3.** Univariate Correlations of Anterior Cingulate ROI with MAIA total score and subscales. | | | | |
| --- | --- | --- | --- | --- |
| **Measure** | **Correlation with ROI** | **95 % CI** | |  |
|  |  | **Lower** | **Upper** |  |
| Total | 0.32 | 0.03 | 0.56 |  |
| Emotion Awareness | 0.32 | 0.02 | 0.56 |  |
| Self-Regulation | 0.28 | -0.02 | 0.53 |  |
| Body Listening | 0.28 | -0.02 | 0.53 |  |
| Trusting | 0.27 | -0.03 | 0.52 |  |
| Attention Regulation | 0.27 | -0.03 | 0.52 |  |
| Noticing | 0.14 | -0.17 | 0.42 |  |
| Not Worrying | 0.12 | -0.19 | 0.40 |  |
| Not Distracting | -0.11 | -0.40 | 0.19 |  |
